# Functional connectivity changes are associated with disability progression in multiple sclerosis: a longitudinal fMRI study

**DOI:** 10.1007/s00415-025-13515-0

**Published:** 2025-11-27

**Authors:** Claudia Piervincenzi, Abhineet Ojha, Silvia Tommasin, Federica Satriano, Nikolaos Petsas, Antonio Gallo, Alessandro d’Ambrosio, Nicola De Stefano, Rosa Cortese, Paola Valsasina, Nicolò Tedone, Carlo Pozzilli, Maria A. Rocca, Massimo Filippi, Patrizia Pantano, the INNI Network, Nicolò Tedone, Costanza Giannì, Elena Barbuti, Loredana Storelli, Stefania Sala, Elisabetta Pagani, Paolo Preziosa, Alvino Bisecco, Riccardo Borgo, Valentina Rippa, Fabrizio Esposito, Maria Laura Stromillo, Riccardo Tappa Brocci, Viola Baione

**Affiliations:** 1 Department of Human Neurosciences, Sapienza University of Rome, Rome, Italy; 2 UniCamillus-Saint Camillus International University of Health Sciences, Rome, Italy; 3 School of Medical Statistics and Biometry, Dept of Public Health and Infectious Diseases, Sapienza University of Rome, Rome, Italy; 4 Department of Advanced Medical and Surgical Sciences, and 3 T MRI‑Center, University of Campania “Luigi Vanvitelli”, Naples, Italy; 5 First Division of Neurology and Neurophysiopathology, AOU Luigi Vanvitelli, Naples, Italy; 6 Department of Medicine, Surgery and Neuroscience, University of Siena, Siena, Italy; 7 Neuroimaging Research Unit, Division of Neuroscience, IRCCS San Raffaele Scientific Institute, Milan, Italy; 8 Neurology Unit, IRCCS San Raffaele Scientific Institute, Milan, Italy; 9 Vita-Salute San Raffaele University, Milan, Italy; 10 Neurorehabilitation Unit, IRCCS San Raffaele Scientific Institute, Milan, Italy; 11 Neurophysiology Service, IRCCS San Raffaele Scientific Institute, Milan, Italy; 12 IRCCS NEUROMED, Pozzilli, IS Italy

**Keywords:** Multiple sclerosis (MS), Magnetic resonance imaging (MRI), Resting-state functional MRI (fMRI)

## Abstract

**Background:**

Resting-state functional connectivity (FC) alterations in people with multiple sclerosis (PwMS) have been hypothesized to reflect either adaptive or maladaptive plasticity. Investigating FC longitudinal evolution and its relationship with disability progression can help clarify this issue. This study examined 5-year FC changes in pwMS and their clinical relevance.

**Methods:**

From the Italian Neuroimaging Network Initiative database, we included 156 pwMS with two clinical visits and 3T-MRI scans acquired on the same scanner 4–6 years apart. Clinical/neuropsychological visits included the Expanded Disability Status Scale (EDSS), Nine-Hole Peg Test (9HPT), Timed 25-Foot Walk Test (T25FWT), Paced Auditory Serial Addition Test (PASAT3), and Symbol Digit Modalities Test (SDMT). One hundred fifty-six age- and sex-matched healthy subjects (HS) with baseline MRI and the same tests were also included. Based on the EDSS, pwMS were divided into three groups: low disability (0–1.5; *N* = 78), mild disability (2–3.5; *N* = 50), and high disability (≥ 4; *N* = 28). Resting-state networks (RSNs) were identified using independent component analysis. Baseline and longitudinal FC changes were correlated with baseline and follow-up clinical/neuropsychological measures.

**Results:**

At baseline, the low-disability group showed significantly higher FC in all RSNs (FDR-corrected *p* < 0.05) compared to HS, which correlated with better baseline scores (SDMT, T25FWT) and less worsening at follow-up (PASAT3, 9HPT). The mild- and high-disability groups exhibited mixed FC abnormalities, with both higher and lower FC than HS in several RSNs. In the mild-disability group, higher FC was associated with worse baseline scores (SDMT, T25FWT) and greater clinical worsening (PASAT3, 9HPT, T25FWT). In the high-disability group, higher sensorimotor baseline FC correlated only with worse baseline 9HPT. Longitudinally, all RSNs showed FC increase in the low-disability group, but a FC decrease in the other groups. FC increases in the low-disability group generally correlated with better clinical outcome (T25FWT), while FC decreases in the mild-disability group correlated with clinical worsening (9HPT, T25FWT).

**Conclusions:**

FC increases appear to reflect compensatory mechanisms in low-disability pwMS, while in more disabled patients, FC alterations likely represent maladaptive responses. These findings support resting-state FC as a biomarker for monitoring disease progression and treatment response in MS.

**Supplementary Information:**

The online version contains supplementary material available at 10.1007/s00415-025-13515-0.

## Introduction

Multiple sclerosis (MS) is a chronic inflammatory and neurodegenerative disease of the central nervous system characterized by demyelination, axonal loss, and progressive disability [1, 2]. In addition to structural damage, alterations in brain functional connectivity (FC), as measured using resting-state functional MRI (fMRI), have been increasingly recognized as relevant to the clinical manifestations of the disease [3–5]. fMRI provides insights into how the brain reorganizes itself in response to MS-related damage, offering potential biomarkers for disease progression and therapeutic targets [3, 4, 6].

In people with MS (PwMS), resting-state FC alterations observed in comparison with healthy subjects (HS) show a complex pattern of increased and decreased connectivity [7–12]. Such alterations have been interpreted as either adaptive mechanisms promoting compensation or maladaptive processes contributing to clinical decline (for reviews see [4, 13, 14]).

Understanding whether FC alterations are adaptive or maladaptive requires studies that examine how FC relates to clinical status over time [4, 15]. To date, few studies have investigated how the FC evolves over time using longitudinal MRI data, usually including a short follow-up period (maximum 2 years) and small sample sizes [16–19]. An even smaller number of studies have included medium-to-long-term follow-up (3–5 years), focusing on cognitive worsening in a small cohort of patients [20] or functional stability [21]. While informative, these studies do not yet allow to draw definitive conclusions regarding the clinical relevance of FC changes over time, and critical knowledge gaps remain in our understanding of functional reorganization in MS. Among these, the interpretation of FC alterations—whether they represent adaptive plasticity or maladaptive processes—remains controversial, partly due to heterogeneous findings and limited longitudinal validation. Moreover, most studies have focused on cognitive outcomes [7, 11, 14, 22, 23], whereas motor performance, despite its clinical relevance, has received considerably less attention. The variability of FC changes across different disease stages is also poorly characterized, as is its relationship with underlying structural damage.

Longitudinal studies are, therefore, crucial for understanding adaptive versus maladaptive plasticity in MS, as they allow monitoring of changes in brain structure, function, and clinical outcomes within the same individuals over time, offering a clearer view of the temporal dynamics underlying disease progression.

To address these issues, the present study investigated FC changes over a 5-year period in a large cohort of PwMS grouped according to baseline disability. By analyzing FC patterns at baseline and follow-up, and exploring their association with motor and cognitive performance, we aimed to clarify the functional relevance of FC changes and assess their potential value as markers of disability progression in MS.

## Materials and methods

### Study design and participants

Demographic, clinical/neuropsychological, and MRI data of PwMS and healthy subjects (HS) were retrospectively retrieved from the Italian Neuroimaging Network Initiative (INNI) repository (https://database.inni-ms.org), which includes data from four Italian Research Centers dedicated to MS research (IRCCS San Raffaele Scientific Institute, Milan; Sapienza University of Rome; University Campania “Luigi Vanvitelli”, Naples; University of Siena), [24].

The study protocols were approved by the local ethics committees and were performed in accordance with the ethical standards laid down in the 1964 Declaration of Helsinki and its later amendments. All PwMS and HS signed a written informed consent form. All data were anonymized to protect the subjects' privacy. The inclusion criteria for the INNI database have been reported elsewhere [24].

To be included in the present study, PwMS had to satisfy the following criteria: availability of two MRI scans acquired with the same scanner, 4–6 years apart; availability of demographic data (age, sex, and years of education), right-handedness, clinical information (disease duration and phenotype), and Expanded Disability Status Scale (EDSS) score; and availability of anatomical three-dimensional (3D) T1‐weighted images and resting-state functional MRI (RS-fMRI). The availability of demographic data and both 3D T1-weighted and RS-fMRI scans were also required for HS.

Other clinical and neuropsychological scales, including Nine-Hole Peg Test (9HPT) using dominant (DH) and non-dominant (NDH) hands, Timed 25-Foot Walk Test (T25FWT), Paced Auditory Serial Addition Test 3 s (PASAT3), and Symbol Digit Modalities Test (SDMT) were also available in the database if collected. For the PASAT3 and SDMT, Z-scores adjusted for age and education were derived using normative data from a sample of 200 healthy Italian adults [25]. Delta values of all the above-mentioned scores were calculated as Δ = (follow-up score − baseline score) and were used for further analysis.

PwMS were divided into three groups according to the EDSS: low disability (EDSS: 0–1.5), mild disability (EDSS: 2–3.5), and high disability (EDSS ≥ 4) [26]; HS were stratified by age and sex to match the patient subgroups, with an equal number of HS included in each group .

### MRI data acquisition

Baseline and follow-up brain MRI scans were obtained using 3.0 T scanners. MRI sequences included 3D T1-weighted, proton‐density/T2‐weighted and/or Fluid Attenuated Inversion Recovery (FLAIR) images and RS-fMRI. Details regarding acquisition protocols are reported in Supplementary Materials (see Supplementary Table 1).

### MRI data analysis

Structural and functional images were pre- and post-processed using fMRIPrep 20.2.3 [27] and the FMRIB Software Library (FSL, version 6.0.7.13) (https://fsl.fmrib.ox.ac.uk/fsl/fslwiki). The structural and functional pipelines are described in the Supplementary Materials.

### Structural MRI measures

#### White matter lesion volume

Focal T2-hyperintense white matter (WM) lesions were previously identified at baseline and follow-up by experienced researchers according to standardized procedures [28] and semi-automatically segmented using a local thresholding segmentation technique (Jim, Xinapse System, Colchester, UK; http://www.xinapse.com). For each subject, T2-lesion volume at baseline and follow-up was calculated using the FSL software package.

#### Global brain volumes

To improve tissue segmentation, 3D T1-weighted images were lesion-filled using the tool available in FSL [29]. Measures of global brain volume and gray matter (GM) volume at baseline were obtained using SIENAx2 [30], while measures of percentage brain volume change (PBVC) between baseline and follow-up were obtained using SIENA, part of FSL.

#### Functional connectivity

Preprocessed RS-fMRI data from all subjects and timepoints were temporally concatenated and submitted to group independent component analysis (ICA) using FSL’s MELODIC tool [31], with a model order of 40 components. This dimensionality was chosen because it allowed the separation of well-known resting-state networks (RSNs), including the basal ganglia network, while maintaining a sufficiently low number of components to support interpretability and adequate spatial segmentation [21, 32, 33]. The RSNs of interest covered the entire brain and were selected via spatial correlation coefficients (*fslcc* tool) using RSNs templates [34, 35], and then verified by expert visual inspection.

The set of spatial maps from the group-average analysis was used to generate subject-specific versions of the spatial maps and associated time series using a dual regression technique [36, 37]. Individual difference maps between T1 and T0 (ΔFC maps) were then obtained for each RSN.

##### Data harmonization

To remove the effects of scanner- and time-related variability, we applied the longitudinal ComBat harmonization method (LongCombat) [38] to both structural and functional data. Resting-state FC was harmonized separately for each selected RSN [39]. Age and sex were included as biological covariates in the harmonization process.

### Statistical analyses

Statistical analyses of demographic, clinical, neuropsychological, and structural MRI parameters were performed using SPSS statistics software (version 22.0). Normality of demographic and clinical data was assessed using the Shapiro–Wilk test.

Between-group differences at baseline between pwMS and HS were tested using the Mann–Whitney *U* test and Chi-square test for continuous and dichotomous variables, respectively (*p* < 0.05). Baseline differences in demographic, clinical, and structural MRI parameters, as well as PBVC, among the PwMS groups were assessed using the Kruskal–Wallis test.

Within each PwMS group, longitudinal changes were assessed using the Wilcoxon signed-rank test (*p* < 0.05).

#### Within-network FC

Subject-specific spatial maps obtained from dual regression analysis were entered into group-level voxelwise analyses. For each RSN, baseline FC differences between each PwMS group and its matched HS group were assessed using two-sample unpaired *t* tests, including age, sex and normalized brain volume as covariates of no interest.

Within-network FC changes over time were assessed using voxelwise one-way ANCOVAs across pwMS groups, adjusting for age, sex, disease duration, and normalized brain volume. Voxelwise statistical analyses were performed with permutation-based non-parametric statistics using the FSL Randomise permutation-based program with 5000 permutations [40].

In pwMS, Randomise tool was also used to examine the statistical correlation between i) baseline FC alterations and baseline behavioral performance, ii) baseline FC alterations and Δscores of behavioral measures and iii) FC longitudinal changes and Δscores of behavioral measures. All results were corrected using false discovery rate (FDR) correction [41] for multiple comparisons (*p* < 0.05). Anatomical localization of significant clusters was established according to the Harvard–Oxford cortical, subcortical and cerebellar structural atlases included in the FMRIB’s Software Library (http://www.fmrib.ox.ac.uk/fsl/data/atlasdescriptions.html).

#### Between-network FC

Between-network FC differences were investigated using the FSLNets toolbox, following standard procedures (http://fsl.fmrib.ox.ac.uk/fsl/fslwiki/FSLNets). Subject-wise correlation matrices of both full and partial correlations of selected RSN time courses were generated and between-subject testing was then conducted across correlation values (Z-transformed) acquired for pairs of independent components.

Between-network connectivity differences at baseline between each PwMS group and its matched HS group were investigated using the FSL Randomise tool (5,000 permutations; age, sex, and normalized brain volume as covariates of no interest). Between-network FC changes over time were assessed using one-way ANCOVAs across pwMS groups on ΔFC values (defined as the difference between follow-up and baseline z-transformed correlation coefficients), adjusting for age, sex, disease duration, and normalized brain volume.

In pwMS, Spearman’s rank correlations were performed to assess the relationship between i) baseline between-network FC alterations and baseline behavioral performance, ii) baseline between-network FC alterations and Δscores of behavioral measures, and iii) between-network FC longitudinal changes and Δscores of behavioral measures. All results were corrected using FDR correction.

## Results

A total of 156 PwMS and 156 HS met the inclusion criteria and were included in the study. Among pwMS, 78 subjects were assigned to the low-disability group (EDSS 0–1.5), 50 to the mild-disability group (EDSS 2–3.5), and 28 to the high-disability group (EDSS ≥ 4). Each PwMS group had an equal number of matched HS (i.e., 78, 50, and 28, respectively).

### Demographic, clinical/neuropsychological and structural MRI data

#### Baseline comparisons

At baseline, the low-disability group showed no significant differences in age and sex but significantly lower education levels (*p* = 0.005) with respect to matched HS. PwMS exhibited slower performance in the 9HPT–NDH (*p* = 0.046) and T25FWT (*p* < 0.001), but they did not significantly differ in the remaining clinical/cognitive measures and structural MRI metrics compared to HS (Table 1).

**Table 1:** Demographic, clinical, neuropsychological, and structural MRI measures in low-disability PwMS and healthy subjects

Low-disability PwMS (EDSS 0–1.5)						
	HS	pwMS baseline	pwMS follow-up	Δ	HS vs. pwMS baseline	pwMS baseline vs. follow-up
Demographic/clinical features					P*	P**
N	78	78	78	–	–	–
Age	36.7 ± 8.9	36.4 ± 8.4	41.5 ± 8.4	–	0.943	–
Female/male, n (%)	61 (78)/17 (22)	61 (78)/17 (22)	–	–	1	–
Education, y	16.5 ± 3.6	14.7 ± 3.1	–	–	**0.005**	–
Disease duration, y	–	6.3 ± 6.2	11.1 ± 6.2	–	–	–
EDSS score, median [range]	–	1.5 [0–1.5]	1.5 [0–4.0]	–	–	**0.001**
Phenotype						
RRMS, n (%)	–	78 (100)	78 (100)	–	–	–
PMS, n (%)	–	0 (0)	0 (0)	–	–	–
Disease-modifying treatment						
None, n (%)	–	29 (36)	12 (15)	–	–	–
Low-efficacy treatment, n (%)	–	39 (50)	50 (64)	–	–	–
High-efficacy treatment, n (%)	–	11 (14)	16 (21)	–	–	–
Clinical/neuropsychological scores						
9HPT dominant hand, s	18.0 ± 2.7 (N = 45)	18.3 ± 2.4 (N = 57)	19.1 ± 2.7 (N = 40)	0.9	0.460	**0.011**
9HPT non-dominant hand, s	19.2 ± 2.7 (N = 45)	20.4 ± 3.0 (N = 57)	20.4 ± 2.9 (N = 40)	0.01	**0.046**	0.994
T25FWT, s	4.9 ± 1.1 (N = 50)	6.3 ± 2.2 (N = 42)	5.9 ± 2.1 (N = 39)	0.1	** < 0.001**	0.309
SDMT, Z	− 0.1 ± 1.5 (N = 27)	− 0.4 ± 1.3 (N = 48)	0.1 ± 1.4 (N = 51)	0.7	0.287	**0.002**
PASAT 3, Z	− 0.1 ± 0.9 (N = 52)	− 0.5 ± 1.1 (N = 66)	− 0.2 ± 1.2 (N = 49)	0.1	0.122	0.299
Structural MRI						
Brain volume (cm^3^)	1,537.9 ± 43.8	1,530.4 ± 51.7		–	0.337	–
Gray matter volume (cm^3^)	849.7 ± 40.3	844.4 ± 43.6		–	0.505	–
T2-lesion volume (cm^3^)		5.2 ± 6.3	6.9 ± 7.9	–	–	** < 0.001**
PBVC, (%)	–	–	− 1.8 ± 1.1	–	–	–

HS = healthy subjects; PwMS = people with Multiple Sclerosis; n = number of subjects; y = years; s = seconds; RRMS = relapsing remitting MS; PMS = progressive MS; EDSS = Expanded Disability Status Scale; 9HPT = Nine-Hole Peg Test; T25FWT = Timed 25-Foot Walk Test; PASAT 3 = Paced Suditory Serial Addition Test with 3.0 seconds interstimulus interval; SDMT = Symbol Digit Modalities Test; PBVC = Percentage brain volume change

Values are reported as mean (standard deviation), if not stated otherwise

*Mann–Whitney *U* test and Chi-square test for continuous and dichotomous variables, respectively (*p* < 0.05)

**Wilcoxon test (*p* < 0.05)

PwMS with mild disability showed no significant differences in age, sex, or education compared to matched HS. PwMS exhibited slower performance in the T25FWT (*p* < 0.001) than HS, whereas no significant differences were found in the other clinical/cognitive measures. They showed lower normalized brain (*p* = 0.019) and GM volumes (*p* = 0.006) than those of HS (Table 2).

**Table 2:** Demographic, clinical, neuropsychological, and structural MRI measures of mild-disability PwMS and healthy subjects

Mild-disability PwMS (EDSS 2–3.5)						
	HS	pwMS baseline	pwMS follow-up	Δ	HS vs. pwMS baseline	pwMS baseline vs. follow-up
Demographic/clinical features					P*	P**
N	50	50	50	–	–	–
Age	41.2 ± 10.7	40.7 ± 9.1	45.7 ± 9.0	–	0.972	–
Female/male, n (%)	36 (72)/14 (28)	36 (72)/14 (28)	-	–	1	–
Education, y	15.2 ± 5.1	13.6 ± 3.0	-	–	0.233	–
Disease duration, y	–	12.7 ± 8.1	17.5 ± 8.6	–	–	–
EDSS score, median [range]	–	2.25 [2–3.5]	3.0 [2–6.5]	–	–	**0.002**
Phenotype						
RRMS, n (%)	–	48 (96)	43 (86)	–	–	–
PMS, n (%)	–	2 (4)	7 (14)	–	–	–
Disease-modifying treatment						
None, n (%)	–	13 (26)	13 (26)	–	–	–
Low-efficacy treatment, n (%)	–	28 (56)	22 (44)	–	–	–
High-efficacy treatment, n (%)	–	9 (18)	15 (30)	–	–	–
Clinical/neuropsychological scores						
9HPT dominant hand, s	18.7 ± 3.7 (N = 30)	19.9 ± 2.9 (N = 26)	25.3 ± 14.0 (N = 25)	5.9	0.077	**0.011**
9HPT non-dominant hand, s	20.5 ± 3.9 (N = 30)	22.2 ± 3.9 (N = 26)	23.5 ± 4.7 (N = 25)	1.3	0.075	0.217
T25FWT, s	4.9 ± 1.1 (N = 29)	6.3 ± 2.2 (N = 26)	5.9 ± 2.1 (N = 24)	− 0.01	** < 0.001**	0.809
SDMT, Z	− 0.4 ± 1.1 (N = 19)	− 0.9 ± 1.5 (N = 37)	− 0.4 ± 1.5 (N = 39)	0.3	0.319	0.060
PASAT 3, Z	− 0.3 ± 1.1 (N = 32)	− 0.6 ± 1.2 (N = 44)	− 0.6 ± 1.3 (N = 39)	− 0.1	0.471	0.650
Structural MRI						
Brain volume (cm^3^)	1,525.8 ± 43.0	1,503.3 ± 54.1		–	**0.019**	–
Gray matter volume (cm^3^)	848.4 ± 45.5	821.7 ± 47.4		–	**0.006**	–
T2-lesion volume (cm^3^)		6.4 ± 6.6	8.4 ± 8.2	–	–	** < 0.001**
PBVC, (%)	–	–	− 2.0 ± 1.5	–	–	–

HS = healthy subjects; PwMS = people with Multiple Sclerosis; n = number of subjects; y = years; s = seconds; RRMS = relapsing remitting MS; PMS = progressive MS; EDSS = Expanded Disability Status Scale; 9HPT = Nine-Hole Peg Test; T25FWT = Timed 25-Foot Walk Testt; PASAT 3 = Paced Auditory Serial Addition Test with 3.0 seconds interstimulus interval; SDMT = Symbol Digit Modalities Test; PBVC = Percentage brain volume change

Values are reported as mean (standard deviation), if not stated otherwise

*Mann–Whitney *U* test and Chi-square test for continuous and dichotomous variables, respectively (*p* < 0.05)

**Wilcoxon test (*p* < 0.05)

Finally, pwMS with high disability showed no significant differences in age, sex, or education compared to matched HS. PwMS exhibited slower performance in 9HPT–DH (*p* = 0.005), 9HPT–NDH (*p* = 0.041), and T25FWT (*p* < 0.001) as well as lower SDMT scores (*p* = 0.012) compared to HS. No significant differences were found between pwMS and HS in normalized brain or gray matter volumes (Table 3).

**Table 3:** Demographic, clinical, neuropsychological, and structural MRI measures of high-disability PwMS and healthy subjects

High-disability PwMS (EDSS > 4)						
	HS	pwMS baseline	pwMS follow-up	Δ	HS vs. pwMS baseline	pwMS baseline vs. follow-up
Demographic/clinical features					P*	P**
N	28	28	28	–	–	–
Age	45.8 ± 10.4	47.5 ± 7.7	52.4 ± 7.7	–	0.611	–
Female/male, n (%)	22 (79)/6 (21)	22 (79)/6 (21)	–	–	1	–
Education, y	15.5 ± 4.2	13.6 ± 4.2	–	–	0.107	–
Disease duration, y	–	15.9 ± 5.5	21.0 ± 5.4	–	–	–
EDSS score, median [range]	–	4.5 [4–8^4^–^8^]	5.75 [4–8^4^–^8^]	–	–	**0.001**
Phenotype						
RRMS, n (%)	–	17 (61)	11 (39)	–	–	–
PMS, n (%)	–	11 (39)	17 (61)	–	–	–
Disease-modifying treatment						
None, n (%)	–	13 (47)	10 (36)	–	–	–
Low-efficacy treatment, n (%)	–	11 (39)	10 (36)	–	–	–
High-efficacy treatment, n (%)	–	4 (14)	8 (28)	–	–	–
Clinical/neuropsychological scores						
9HPT dominant hand, s	18.8 ± 2.7 (N = 19)	27.1 ± 15.3 (N = 16)	32.0 ± 18.5 (N = 18)	7.2	**0.005**	0.158
9HPT non-dominant hand, s	21.3 ± 2.7 (N = 19)	24.7 ± 5.3 (N = 16)	27.4 ± 5.6 (N = 17)	2.7	**0.041**	**0.030**
T25FWT, s	4.5 ± 1.4 (N = 16)	10.2 ± 5.4 (N = 12)	9.4 ± 2.7 (N = 11)	1.8	** < 0.001**	0.310
SDMT, Z	− 0.3 ± 1.1 (N = 16)	− 1.3 ± 1.3 (N = 18)	− 1.4 ± 1.2 (N = 22)	0.1	**0.012**	0.610
PASAT 3, Z	− 0.7 ± 1.2 (N = 20)	− 0.6 ± 1.3 (N = 22)	− 0.7 ± 1.1 (N = 22)	− 0.02	0.880	0.623
Structural MRI						
Brain volume (cm^3^)	1,518.3 ± 47.1	1,493.1 ± 47.3		–	0.091	–
Gray matter volume (cm^3^)	830.1 ± 37.9	808.6 ± 44.5		–	0.095	–
T2-lesion volume (cm^3^)		12.2 ± 12.7	15.7 ± 16.9	–	–	**0.002**
PBVC, (%)	–	–	− 2.1 ± 1.4	–	–	–

HS = healthy subjects; PwMS = people with Multiple Sclerosis; n = number of subjects; y = years; s = seconds; RRMS = relapsing remitting MS; PMS = progressive MS; EDSS = Expanded Disability Status Scale; 9HPT = Nine-Hole Peg Test; T25FWT = Timed 25-Foot Walk Test; PASAT 3 = Paced Auditory Serial Addition test with 3.0 seconds interstimulus interval; SDMT = Symbol Digit Modalities Test; PBVC = Percentage brain volume change

Values are reported as mean (standard deviation), if not stated otherwise

*Mann–Whitney *U* test and Chi-square test for continuous and dichotomous variables, respectively (*p* < 0.05)

**Wilcoxon test (*p* < 0.05)

When comparing the three pwMS groups, we observed significant differences in age and disease duration (both *p* < 0.001) at baseline (Supplementary Table 2). Performance on the 9HPT–DH (*p* < 0.001) and 9HPT–NDH (*p* = 0.007), T25FWT (*p* < 0.001), and SDMT (*p* = 0.031), but not PASAT3 scores, also differed between patients’ groups (Supplementary Table 2). Significant differences among pwMS groups were also observed in normalized brain volume, GM volume and T2-lesion volume (all *p* = 0.001) (Supplementary Table 2).

#### Longitudinal changes in pwMS

In the entire group of pwMs, the follow-up duration had a median of 5.1 years (IQR = 1.0). No significant difference in follow-up duration was found between patient groups (Supplementary Table 2). All groups showed a significant worsening in EDSS over time (Tables 1, 2 and 3).

PwMS with low disability showed significant motor worsening, with slower performance on the 9HPT–DH (*p* = 0.011), alongside an improvement in SDMT scores (*p* = 0.002). T2-lesion volume increased over time (*p* < 0.001) (Table 1).

PwMS with mild disability showed significant worsening in the 9HPT–DH performance (*p* = 0.011), whereas no significant changes were observed in other motor or cognitive scores. A significant increase in T2-lesion volume was also observed (*p* < 0.001) (Table 2).

PwMS with high disability showed significant worsening in the 9HPT–NDH performance (*p* = 0.030), while no significant changes were observed in the other motor or cognitive scores. T2-lesion volume also increased over time (*p* = 0.002) (Table 3).

No significant differences in PBVC were observed among the groups (Supplementary Table 2).

### Resting-state FC

From ICA analysis, we identified 11 components that showed the highest spatial correlation coefficients with RSN templates: auditory (AUN), basal ganglia (BGN), cerebellar (CBN), dorsal attention (DAN), default mode (DMN), executive control (ECN), left and right frontoparietal (lFPN and rFPN), lateral visual (LVN), medial visual (MVN), and sensorimotor (SMN) networks (Supplementary Fig. 1).

#### Baseline FC comparisons

**Fig. 1:**
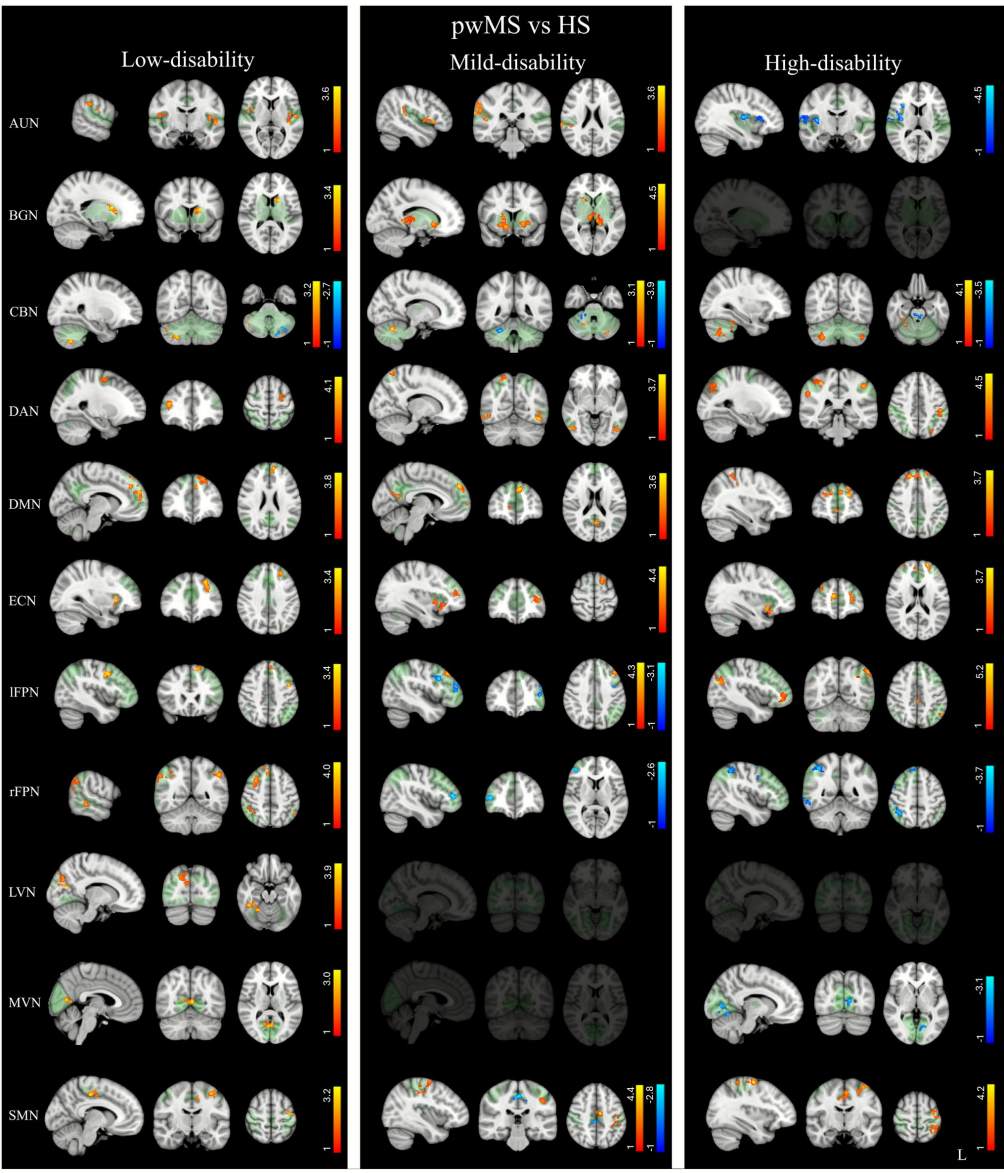
Resting-state networks (RSNs) showing significant within-network functional connectivity differences between people with Multiple Sclerosis (pwMS) groups and corresponding healthy subjects (HS) (*p* < 0.05, FDR corrected). Results for each RSN are overlaid onto the corresponding network (green) in the MNI152 standard brain. Red–yellow and blue–light-blue colors indicate areas of higher and lower FC in pwMS compared with HS, respectively. Color bars represent *t* values

Baseline within-network FC comparisons with HS showed significant FC alterations across all patients’ groups (*p* < 0.05 FDR-corrected). Specifically, pwMS with low-disability exhibited higher FC than HS in all RSNs (Fig. 1, Supplementary Table 3), which correlated negatively with baseline motor tests (LVN with T25FWT) and positively with baseline cognitive tests (rFPN with SDMT), indicating that the higher the FC, the better the baseline performance (Supplementary Fig. 2, Supplementary Table 4). In this group, baseline FC also correlated negatively with Δscores of motor tests (AUN and LVN with Δ9HPT–DH) and positively with Δscores of cognitive tests (BGN with ΔPASAT3), indicating that higher baseline FC was associated with less clinical worsening (Supplementary Fig. 3, Supplementary Table 5).

The mild-disability group exhibited clusters of both higher and lower FC than the HS in several RSNs (Fig. 1, Supplementary Table 3). In this group, baseline FC correlated positively with baseline motor performance (DMN with T25FWT) and negatively with baseline cognitive performance (AUN with SDMT), indicating that, unlike in the low-disability group, higher baseline FC was associated with worse baseline motor and cognitive performance (Supplementary Fig. 2, Supplementary Table 4). In this group, higher baseline FC also correlated positively with Δscores of motor tests (BGN and lFPN with Δ9HPT–DH; BGN with ΔT25FWT) and negatively with Δscores of cognitive tests (BGN and DMN with ΔPASAT3), indicating that higher baseline FC was associated with greater clinical worsening (Supplementary Fig. 3, Supplementary Table 5).

Finally, in the high-disability group, we found both higher and lower FC clusters in various RSNs (Fig. 1, Supplementary Table 3). In this group, baseline FC increases in the SMN correlated with higher baseline 9HPT–DH, indicating that a higher FC in this network was associated with poorer dominant-hand fine motor performance (Supplementary Fig. 2, Supplementary Table 4). No correlations were found between baseline FC and Δscores of motor and cognitive tests.

No correlations were observed in any group between areas of reduced FC and clinical tests.

Regarding between-network FC, no significant differences were observed between HS and pwMS with low or mild disability. Conversely, pwMS with high disability exhibited significantly higher partial correlation values between the BGN and LVN compared to HS (mean Z correlation values of − 0.55 and − 1.25, respectively), indicating a reduction in the strength of the anticorrelation of these RSNs. However, BGN–LVN correlation values did not correlate with clinical scores at baseline or Δ clinical scores.

#### Longitudinal FC changes in pwMS

**Fig. 2:**
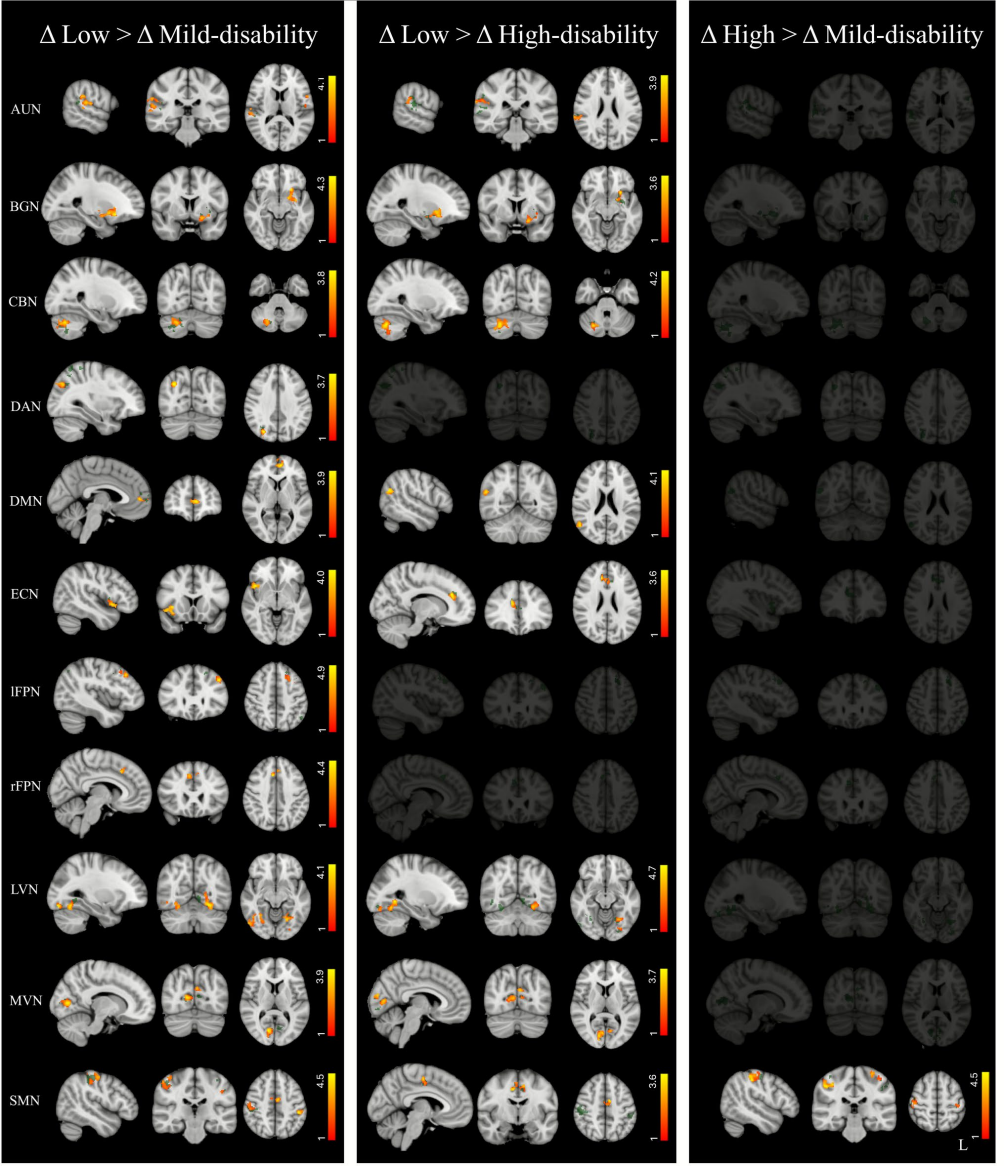
Resting-state networks (RSNs) showing significant group differences in longitudinal changes of functional connectivity (ΔFC = follow-up—baseline) among people with Multiple Sclerosis (pwMS) with low, mild, and high disability. Results for each RSN (*p* < 0.05, FDR-corrected) are overlaid onto the corresponding F-map (shown in green) in the MNI152 standard brain. Red–yellow color indicates areas where ΔFC was higher in one group relative to another, respectively. Color bars represent *t* values

**Fig. 3:**
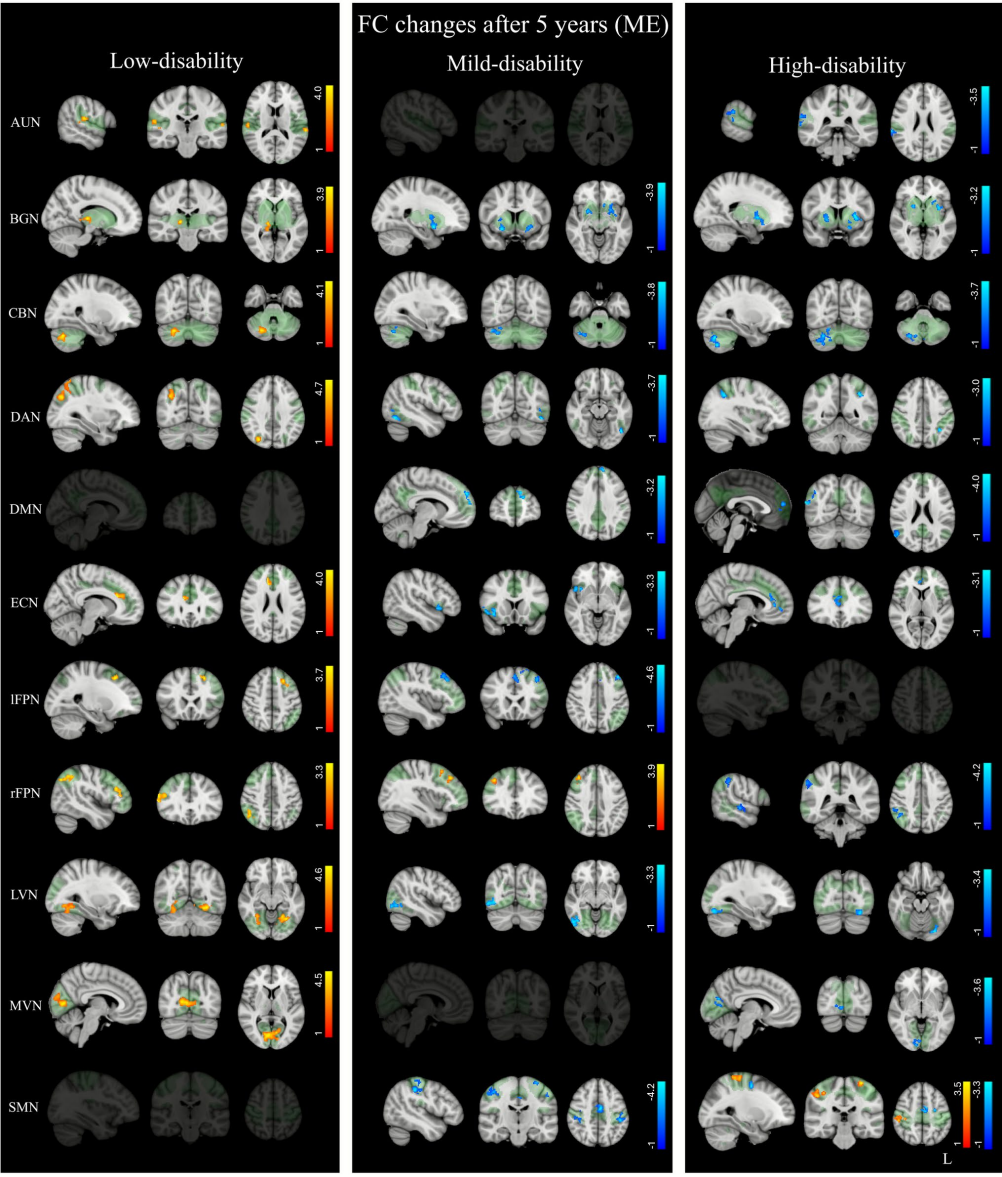
Voxel-wise main effects (ME) from the ANCOVA model showing significant longitudinal FC changes within resting-state networks (RSNs) for each disability group (*p* < 0.05, FDR corrected). Results for each RSN are overlaid onto the corresponding network (green) in the MNI152 standard brain. Red–yellow and blue–light-blue color bars indicate areas of FC increases and decreases, respectively. Color bars represent *t* values

The ANCOVA analysis revealed significant group effects in most RSNs (see F-maps in Fig. 2, in green). Post-hoc comparisons showed that ΔFC values were significantly higher in pwMS with low disability compared with both mild- and high-disability groups, whereas no differences were observed between the mild- and high-disability groups except for the sensorimotor network, where ΔFC was higher in pwMS with high disability (Fig. 2, Supplementary Table 6). The group-wise main effects showed that over time, pwMS with low disability exhibited FC increases, while mild- and high-disability groups showed FC decreases (Fig. 3, Supplementary Table 7).

In the low-disability group, FC increments within the DAN were positively correlated with Δ9HPT–DH, whereas FC increments in the MVN were correlated with Δ9HPT–NDH. Conversely, FC increments in the DAN, LVN, and MVN were negatively correlated with gait performance (ΔT25FWT) (Supplementary Fig. 4, Supplementary Table 8). These results suggest that a further increase in FC over time is associated with worsening of hand fine motor performance, but it is also associated with more stable gait function.

In the mild-disability group, FC decrements in the CBN and ECN were negatively correlated with changes in motor scores (Δ9HPT–NDH for CBN and ΔT25FWT for CBN and ECN) (Supplementary Fig. 4, Supplementary Table 7). These results suggest that greater FC reduction is associated with greater decline in motor performance. In addition, in this group, we observed a positive correlation between PBVC and FC decrements in the SMN, indicating that the higher the atrophy rate, the greater the FC decline (Supplementary Fig. 4, Supplementary Table 7).

Finally, in the high-disability group, no significant correlations were found between FC decrements and Δ scores of clinical tests. However, a positive correlation was found between the increase in SMN FC and Δ9HPT–DH, indicating that as FC increases, dominant-hand fine motor performance worsens (Supplementary Fig. 4, Supplementary Table 7).

Regarding between-network FC longitudinal changes, the ANCOVA analysis did not reveal any significant group effects.

## Discussion

In this longitudinal study, we investigated resting-state FC changes over a 5-year period in a large cohort of PwMS, grouped according to baseline disability, to evaluate whether FC alterations reflect adaptive or maladaptive mechanisms in relation to disability severity and progression.

Baseline FC patterns of pwMS were first compared to those of matched HS, and longitudinal FC changes were then assessed across pwMS groups, along with their associations with motor and cognitive outcomes.

PwMS with low disability showed higher FC at baseline across all investigated RSNs compared to HS, and further FC increases over time. These FC enhancements were significantly associated with better baseline cognitive and motor performance and more favorable clinical outcomes. Conversely, pwMS with mild and high disability exhibited a mixed pattern of FC alterations at baseline compared to HS and overall FC reductions over time, which were generally related to worsening motor outcomes.

These findings highlight the complexity of functional reorganization in MS, suggesting that the clinical relevance of FC alterations depends on disease stage. They also support the utility of resting-state FC as a biomarker for monitoring disease progression and assessing treatment efficacy in PwMS.

In this context, this study extends previous longitudinal investigations of adaptive–maladaptive FC dynamics [11, 16, 20, 21] by encompassing a longer follow-up and a broader, multicenter cohort, with systematic evaluation of both motor and cognitive outcomes.

### Longitudinal FC changes reflect adaptive and maladaptive dynamics according to disease stage

To better interpret FC alterations in the context of disease progression, PwMS were stratified into three groups (low, mild, and high disability). This subdivision also reflected progressive increases in age, disease duration, and brain structural damage, suggesting that the three groups represent not only different disability levels but also distinct stages of the disease.

Specifically, PwMS with low disability showed higher FC at baseline across nearly all RSNs compared to HS, along with further FC increases over time, both of which were significantly associated with better clinical performance and less disability progression over time, supporting the idea of a beneficial role of functional reorganization in the early stages of MS [11, 16, 20, 42].

Conversely, PwMS with mild disability exhibited a more heterogeneous pattern at baseline, with areas of both higher and lower FC compared to HS. Notably, higher FC in this group was associated not only with worse motor and cognitive performance at baseline but also with greater clinical worsening over time, suggesting that, at this stage, FC increases may already reflect a shift from adaptive reorganization toward inefficient or maladaptive processes [11, 13, 43, 44]. Longitudinally, this group showed widespread FC reductions across all RSNs, which significantly correlated with clinical worsening.

Finally, PwMS with high disability also showed a mixed FC pattern at baseline, with higher FC in the SMN associated with poorer dominant-hand fine motor performance, supporting the idea that in advanced stages, higher FC may reflect maladaptive or pathological overactivation [22, 45]. In this group, longitudinal data revealed FC decreases in most RSNs, although no clear correlations with clinical changes were observed. In the sensorimotor network, however, FC increased, and was associated with motor decline indicating that the concept of maladaptive reorganization, hypothesized for cognitive function, [4, 14, 44], may also be applicable to sensorimotor function. Although these results are consistent with maladaptive reorganization, the high-disability subgroup was smaller and clinically heterogeneous; therefore, subgroup-specific findings should be interpreted with caution and need to be replicated in larger, more homogeneous samples.

While most of our findings revealed relevant within-network FC alterations, few between-network FC connectivity changes were observed. At baseline, only PwMS with high disability showed a significant reduction in the anticorrelation between the basal ganglia and lateral visual networks. These alterations did not correlate with clinical performance; therefore, their clinical significance remains unclear.

The present findings demonstrate that FC alterations in MS are not univocal and should be interpreted in the context of the disease stage and clinical status [3, 4]. Our longitudinal design clearly indicates that the same direction of FC change (e.g., an increase) can be either beneficial or detrimental depending on the clinical and network context, as previously hypothesized [4, 17, 45] and observed, although in studies with shorter follow-up periods [16] or using modeling approaches [44].

### Functional connectivity and motor system reorganization

Although resting-state FC alterations have been extensively investigated in relation to cognitive dysfunction in pwMS [7, 11, 14, 22, 23, 42], the relevance of FC changes in motor performance has received less attention [4, 10, 13].

This study addressed this gap by examining both baseline and longitudinal FC–motor associations across disease stages, providing a multicenter, 5-year longitudinal characterization of motor-network reorganization alongside cognitive measures. We found that FC changes impact distinct aspects of motor performance in different ways, depending on the disease stage and the specific motor domain involved. Motor performance, particularly manual dexterity, showed distinct FC–behavior associations across disability stages, reflecting stage-specific shifts in functional reorganization.

In the low-disability group, longitudinal FC increases in networks such as the dorsal attention, medial, and lateral visual were associated with gait stability, suggesting effective compensatory plasticity. However, increased FC also correlated with worsened dominant-hand 9HPT, the only motor test that worsened over time, indicating that early FC increases may not always reflect adaptive responses and that this specific function may be particularly vulnerable to early pathological changes and inefficient network reorganization [16, 45–47]. While certain functional networks may still possess sufficient reserve capacity to reorganize efficiently, others may already show early signs of functional failure, possibly due to baseline task demands or lateralization effects, such as the greater reliance on the dominant hand in daily activities, which has been shown to be particularly sensitive to early performance decline [46, 47].

In the mild-disability group, the pattern shifted toward a more generalized inefficiency. Here, higher baseline FC in the frontoparietal and basal ganglia networks was associated with poorer 9HPT performance, particularly in the dominant hand. Over time, this group exhibited widespread FC decreases across multiple networks, which were significantly associated with further decline not only in manual dexterity but also in gait performance; specifically, FC reductions in the cerebellar and executive networks correlated with worsening in the T25FWT and 9HPT. These parallel changes in upper and lower limb function suggest that, unlike in the early disease phase, compensatory mechanisms are globally exhausted in this phase, resulting in functional decline across multiple motor subsystems. This selective vulnerability may reflect the cumulative burden of disease on more demanding or lateralized motor functions after a decade of MS progression [13, 48].

Finally, in the high-disability group, maladaptive patterns became more anatomically specific. Residual FC increases in the left primary motor cortex were significantly associated with worse baseline 9HPT–DH scores. These findings reinforce the interpretation of pathological overactivation, in which persistent FC increases in core motor areas may no longer reflect successful compensation but rather inefficient or disorganized recruitment, possibly reflecting the collapse of both structural and functional reserve mechanisms [4, 45, 48]. Longitudinally, further FC increases in the right primary motor cortex were significantly associated with worsening performance on the 9HPT of the dominant hand, suggesting that persistent hyperconnectivity within core motor regions may indicate aberrant reorganization within motor networks. This ipsilateral FC increase may represent a failure to maintain proper lateralized functional architecture, potentially due to impaired transcallosal inhibitory control from the dominant hemisphere [49–52], although this hypothesis should be further verified in larger patient samples.

### Link between structural damage and functional connectivity

To better understand how FC changes relate to underlying brain alterations, we also examined structural MRI measures across the patients’ groups. As expected, both brain and GM volumes were progressively reduced from the low- to high-disability group, while T2-lesion volume increased, in line with prior studies [2, 28, 53].

However, the PBVC, a marker of global brain atrophy over time, did not differ significantly between the groups, suggesting that the rate of atrophy progression remains relatively stable across disease stages, in keeping with prior longitudinal studies in MS [54, 55]. Thus, while patients with higher disability exhibited more severe structural damage at the group level, this likely reflects the cumulative effect of disease duration rather than an acceleration of the atrophy rate.

Finally, in the mild-disability group, we observed a modest but significant correlation between PBVC and longitudinal FC reductions in the sensorimotor network. We may speculate that widespread tissue loss could begin to compromise network integrity when compensatory mechanisms begin to fail [4, 44, 48]. However, given that PBVC represents a global measure of atrophy and regional effects were not assessed, this finding should be interpreted with caution and considered exploratory.

### Limitations

The present study is not without limitations. First, direct comparisons of baseline FC between PwMS groups were not performed due to significant differences in demographic/clinical variables (e.g., age, disease duration, performance), which may act as confounding factors; group analyses were focused on longitudinal trajectories rather than group-level FC differences per se. Second, although information on treatment categories was collected, treatment history and switching patterns were not available; therefore, the potential impact of disease-modifying therapies on FC dynamics and clinical progression remains unaccounted. Third, resting-state fMRI was acquired at only two timepoints, limiting our ability to capture nonlinear or dynamic changes in FC trajectories over time. In addition, the high-disability group was smaller and clinically heterogeneous, reducing statistical power to detect FC–clinical associations in this group. Finally, some of the identified correlations involve parameters that did not exhibit significant group-level changes over time (e.g., PASAT3), despite individual variability, which may complicate the interpretation of their clinical relevance. Nevertheless, these findings remain of potential interest, as they may capture inter-individual variability and reveal meaningful associations even when changes in clinical tests do not emerge at the group level.

## Conclusion

The present study supports the potential of resting-state FC as a predictive biomarker in MS. The progression of FC changes across low-to-high-disability groups highlights the association between disease stage and brain functional changes, with increased FC in early stages appearing to support adaptive plasticity and predict better outcomes, while reductions or maladaptive increases in later stages are associated with clinical decline.

Correlations between longitudinal FC changes and concurrent clinical worsening further support the functional relevance of longitudinal FC dynamics, providing additional insight into the ongoing processes of adaptation or failure across disease stages.

When analyzed within clinically defined subgroups, FC measures may serve as prognostic indicators, monitoring tools and guides for therapeutic strategies in MS.

## Supplementary Information

Below is the link to the electronic supplementary material.

Supplementary file1 (DOCX 7193 KB)

## Data Availability

The data that support the findings of this study are available on request from the corresponding author. The data are not publicly available due to privacy or ethical restrictions.
