# Supplementary material for: Functional connectivity changes are associated with disability progression in multiple sclerosis: a longitudinal fMRI study
Source: J Neurol. 2025 Nov 27;272(12):787. doi: 10.1007/s00415-025-13515-0 (PMC12660332; doi:10.1007/s00415-025-13515-0)
Supplement: Supplementary file 1 — Supplementary file1 (DOCX 7193 KB) [file 415_2025_13515_MOESM1_ESM.docx]

**Supplementary Materials.**

# **Title: Functional Connectivity Changes are associated with Disability Progression in Multiple Sclerosis: A Longitudinal fMRI Study**

# **MRI analysis**

*Data preprocessing*

Data preprocessing was performed using *fMRIPrep* 20.2.3 [[1]](https://www.zotero.org/google-docs/?baltI4); RRID:SCR_016216), which is based on *Nipype* 1.5.0 ([[2, 3]](https://www.zotero.org/google-docs/?fH3EYT); RRID:SCR_002502).

Each of the T1-weighted (T1w) images were preprocessed with the following pipeline: first, each of the T1-weighted (T1w) image was corrected for intensity non-uniformity (INU) with N4BiasFieldCorrection [[4]](https://www.zotero.org/google-docs/?g1ONmz), distributed with ANTs 2.3.3 ([[5]](https://www.zotero.org/google-docs/?Vjp4Ua), RRID:SCR_004757), and used as T1w-reference throughout the workflow. The T1w-reference was then skull-stripped with a Nipype implementation of the antsBrainExtraction.sh workflow (from ANTs), using OASIS30ANTs as target template. Brain tissue segmentation of cerebrospinal fluid (CSF), white-matter (WM) and gray-matter (GM) was performed on the brain-extracted T1w using fast (FSL 5.0.9, RRID:SCR_002823, [[6]](https://www.zotero.org/google-docs/?WNHLRF)). Volume-based spatial normalization to two standard spaces (MNI152NLin2009cAsym, MNI152NLin6Asym) was performed through nonlinear registration with antsRegistration (ANTs 2.3.3), using brain-extracted versions of both T1w reference and the T1w template. The following templates were selected for spatial normalization: ICBM 152 Nonlinear Asymmetrical template version 2009c [[[7]](https://www.zotero.org/google-docs/?4sWWBL), RRID:SCR_008796; TemplateFlow ID: MNI152NLin2009cAsym], FSL’s MNI ICBM 152 non-linear 6th Generation Asymmetric Average Brain Stereotaxic Registration Model [[[8]](https://www.zotero.org/google-docs/?B8qGKw), RRID:SCR_002823; TemplateFlow ID: MNI152NLin6Asym].

Functional preprocessing was also performed using *fMRIPrep*. For each subject, the following preprocessing was performed: first, a reference volume and its skull-stripped version were generated using a custom methodology of *fMRIPrep*. Susceptibility distortion correction (SDC) was omitted. The BOLD reference was then co-registered to the T1w reference using flirt (FSL 5.0.9, [[9]](https://www.zotero.org/google-docs/?4EVOAX)) with the boundary-based registration [[10]](https://www.zotero.org/google-docs/?8EneHw) cost-function. Co-registration was configured with nine degrees of freedom to account for distortions remaining in the BOLD reference. Head-motion parameters with respect to the BOLD reference (transformation matrices, and six corresponding rotation and translation parameters) are estimated before any spatiotemporal filtering using mcflirt (FSL 5.0.9, [[11]](https://www.zotero.org/google-docs/?AG4diY)). BOLD runs were slice-time corrected using 3dTshift from AFNI 20160207 ([[12]](https://www.zotero.org/google-docs/?RtdGP0), RRID:SCR_005927). The BOLD time-series (including slice-timing correction when applied) were resampled onto their original, native space by applying the transforms to correct for head-motion. These resampled BOLD time-series will be referred to as preprocessed BOLD in original space, or just preprocessed BOLD. The BOLD time-series were resampled into standard space, generating a preprocessed BOLD run in MNI152NLin2009cAsym space. First, a reference volume and its skull-stripped version were generated using a custom methodology of fMRIPrep. Automatic removal of motion artifacts using independent component analysis (ICA-AROMA, [[13]](https://www.zotero.org/google-docs/?2HZZr5)) was performed on the preprocessed BOLD on MNI space time-series after removal of non-steady state volumes and spatial smoothing with an isotropic, Gaussian kernel of 6mm FWHM (full-width half-maximum). Corresponding “non-aggresively” denoised runs were produced after such smoothing. Additionally, the “aggressive” noise-regressors were collected and placed in the corresponding confounds file. Functional preprocessed data were finally subjected to WM and CSF signal regression and high-pass filtering (100-seconds cut-off) [[13]](https://www.zotero.org/google-docs/?RFdyjr).

# **Supplementary Tables**

**Supplementary Table 1:** MRI acquisition parameters for each center participating in INNI.

|  | **Center A** | | | **Center B** | | | **Center C** | | | **Center D** | | |
| --- | --- | --- | --- | --- | --- | --- | --- | --- | --- | --- | --- | --- |
| MR scanner | Philips medical system—intera | | | GE medical system signa HDxt | | | Siemens Verio | | | Philips medical system—achieva | | |
| MR imaging sequence | Dual-echo | T_1_-weighted TFE | T2*-weighted single-shot EPI | T_2_-weighted FLAIR | T_1_-weighted IR–FSPGR | T2*-weighted gradient-echo EPI | Dual-echo | T_1_-weighted MPRAGE | T2*-weighted single-shot EPI | Dual-echo | T_1_-weighted FFE | T2*-weighted EPI |
| Coil | 8-channel head coil | | | 8-channel head coil | | | 12-channel head coil | | | 32-channel head coil | | |
| Imaging plane | Axial | Axial | Axial | Axial | Sagittal | Axial | Axial | Sagittal | Axial | Axial | Axial | Axial |
| Acquisition voxel [mm^3^] | 1 × 1 × 3 | 1 × 1 × 1 | 1.87 x 1.87 x 4 | 1 × 1 × 3 | 1 × 1 × 1.2 | 4 x 4 x 4 | 1 × 1 × 4 | 1 × 1 × 1 | 3 x 3 x 3 | 1 × 1 × 3 | 1 ×1 × 1 | 1.87 x 1.87 x 4 |
| FOV [mm^2^] | 243 × 243 | 230 × 230 | 240 x 240 | 256 × 256 | 256 × 256 | 256 × 256 | 220 × 220 | 256 × 256 | 192 x 192 | 240 × 240 | 256 × 256 | 234 x 234 |
| TR [ms] | 2910 | 25 | 3000 | 9002 | 6.988 | 1508 | 5310 | 1900 | 3000 | 4000 | 10 | 3000 |
| TE [ms] | 16–80 | 4.6 | 35 | 120 | 2.85 | 32 | 10–103 | 2.9 | 30 | 15–100 | 3.9 | 35 |
| TI [ms] | – | – | - | 2500 | 650 | - | – | 900 | - | – | 900 | - |
| FA [°] | 90 | 30 | 90 | 90 | 8 | 90 | 150 | 9 | 89 | 90 | 8 | 90 |
| ETL | 6 | 1 | - | 1 | 1 | - | 6 | 1 | - | 4 | 128 | 73 |
| TA [min] | 2.43 | 2.75 | 10 | 2.02 | 1.9 | 6.032 | 2.92 | 3.07 | 7 | 8.6 | 8.2 | 10 |
| N vol | - | - | 200 | - | - | 240 | - | - | 140 | - | - | 200 |

INNI: Italian Neuroimaging Network Initiative; Center A: San Raffaele Scientific Institute, Milan; Center B: University Campania “Luigi Vanvitelli”, Naples; Center C: Sapienza University of Rome, Rome; Center D: University of Siena, Siena; FOV: field of view; TR: repetition time; TE: echo time; TI: inversion time; FA: flip angle; ETL: echo train length; TA: acquisition time; TFE: turbo field echo; IR: inversion recovery; FSPGR: fast spoiled gradient echo; MPRAGE: magnetization prepared gradient echo; FFE fast field echo.

**Supplementary Table 2.** Baseline differences in demographic, clinical, and structural MRI measures, as well as PBVC, among PwMS groups (low-, mild-, and high-disability groups).

|  | **pwMS**  **EDSS 0-1.5**  **N=78** | **pwMS**  **EDSS 2-3.5**  **N=50** | **pwMS**  **EDSS ≥4**  **N=28** | ***P**** | **H** | **Post hoc** |
| --- | --- | --- | --- | --- | --- | --- |
| **Demographic/clinical features** |  |  |  |  |  |  |
| Follow-up, y [median (IQR)] | 5.2 [0.9] | 5.1 [1.2] | 4.9 [0.8] | 0.189 | 3.33 | - |
| Age | 36.4 ± 8.4 | 40.7 ± 9.1 | 47.5 ± 7.7 | **<0.001** | 28.45 | low-mild p=**0.024** |
|  |  |  |  |  |  | low-high p<**0.001** |
|  |  |  |  |  |  | mild-high p=**0.013** |
| Female/male, n (%) | 61 (78) / 17 (22) | 36 (72) / 14 (28) | 22 (79) / 6 (21) | 0.688 | 0.75 | - |
| Education, y | 14.7 ± 3.1 | 13.6 ± 3.0 | 13.6 ± 4.2 | 0.135 | 4.00 | - |
| Disease duration, y | 6.3 ± 6.2 | 12.7 ± 8.1 | 15.9 ± 5.5 | **<0.001** | 43.41 | low-mild p<**0.001** |
|  |  |  |  |  |  | low-high p<**0.001** |
|  |  |  |  |  |  | mild-high p=0.157 |
| **Clinical/neuropsychological scores** |  |  |  |  |  |  |
| 9HPT dominant hand, s | 18.3 ± 2.4  (N=57) | 19.9 ± 2.9  (N=26) | 27.1 ± 15.3  (N=16) | **<0.001** | 16.36 | low-mild p=0.081 |
|  |  |  |  |  |  | low-high p<**0.001** |
|  |  |  |  |  |  | mild-high p=0.187 |
| 9HPT non-dominant hand, s | 20.4 ± 3.0  (N=57) | 22.2 ± 3.9  (N=26) | 24.7 ± 5.3  (N=16) | **0.007** | 10.00 | low-mild p=0.189 |
|  |  |  |  |  |  | low-high p=**0.008** |
|  |  |  |  |  |  | mild-high p=0.565 |
| T25FWT, s | 6.3 ± 2.2  (N=42) | 6.3 ± 2.2  (N=26) | 10.2 ± 5.4  (N=12) | **<0.001** | 16.01 | low-mild p=**0.022** |
|  |  |  |  |  |  | low-high p=**0.001** |
|  |  |  |  |  |  | mild-high p=0.422 |
| SDMT, Z | -0.4 ± 1.3  (N=48) | -0.9 ± 1.5  (N=37) | -1.3 ± 1.3  (N=18) | **0.031** | 6.92 | low-mild p=0.846 |
|  |  |  |  |  |  | low-high p=**0.026** |
|  |  |  |  |  |  | mild-high p=0.264 |
| PASAT 3s, Z | -0.5 ± 1.1  (N=66) | -0.6 ± 1.2  (N=44) | -0.6 ± 1.3  (N=22) | 0.945 | 0.11 | - |
| **Structural MRI** |  |  |  |  |  |  |
| Brain volume (cm^3^) | 1,530.4 ± 51.7 | 1,503.3 ± 54.1 | 1,493.1 ± 47.3 | **0.001** | 13.63 | low-mild p=**0.012** |
|  |  |  |  |  |  | low-high p=**0.005** |
|  |  |  |  |  |  | mild-high p=1 |
| Gray matter volume (cm^3^) | 844.4 ± 43.6 | 821.7 ± 47.4 | 808.6 ± 44.5 | **0.001** | 14.70 | low-mild p=**0.016** |
|  |  |  |  |  |  | low-high p=**0.002** |
|  |  |  |  |  |  | mild-high p=0.910 |
| T2-lesion volume (cm^3^) | 5.2 ± 6.3 | 6.4 ± 6.6 | 12.2 ± 12.7 | **0.001** | 14.78 | low-mild p=0.316 |
|  |  |  |  |  |  | low-high p<**0.001** |
|  |  |  |  |  |  | mild-high p=0.060 |
| PBVC, (%) | -1.8 ± 1.1 | -2.0 ± 1.5 | -2.1 ± 1.4 | 0.830 | 0.37 | - |
|  |  |  |  |  |  |  |

PwMS=people with Multiple Sclerosis; n=number of subjects; y=years; s=seconds; IQR=interquartile range; 9HPT=nine-hole peg test; T25FWT=timed 25 feet walking test; PASAT 3/PASAT 2=paced auditory serial addition test with 3.0 seconds interstimulus interval; SDMT=symbol digit modalities test; PBVC= Percentage brain volume change.

Values are reported as average (standard deviation), if not stated otherwise.

* Kruskal–Wallis and Chi-square test for continuous and dichotomous variables, respectively (p <0.05) (mean ± standard deviation, *p*, H, and post hoc test are displayed).

**Supplementary Table 3.** Resting-state networks showing significant within-network functional connectivity differences between people with Multiple Sclerosis (pwMS) groups and healthy subjects (HS) (p <0.05, FDR corrected, minimum cluster extent set at 100 voxels). Peak Montreal Neurological Institute (MNI) coordinates (mm) within clusters were identified using the minimum peak distance between the local maxima of 20 mm. Anatomical localizations of peak MNI coordinates were established according to Harvard-Oxford cortical and subcortical structural atlases and the cerebellar atlas included in FMRIB’s Software Library.

|  |  | **MNI coordinates** | | |  |
| --- | --- | --- | --- | --- | --- |
| **Cluster size (voxels)** | **T** | **x** | **y** | **z** | **Cluster location (local maxima)** |
| **Low-disability > HS** | | | | | |
| **AUN** |  |  |  |  |  |
| 343 | 3.59 | -52 | -12 | 2 | Left Heschl's Gyrus (includes H1 and H2) |
| 143 | 3.08 | 42 | 4 | 8 | Right Central Opercular Cortex |
| 112 | 3.0 | 64 | -30 | 32 | Right Supramarginal Gyrus, anterior division |
| **BGN** |  |  |  |  |  |
| 183 | 3.44 | -18 | 12 | 14 | Left Caudate |
| **CBN** |  |  |  |  |  |
| 150 | 3.20 | 46 | -44 | -34 | Right Cerebellar Crus I |
| 104 | 3.01 | 26 | -60 | -48 | Right Cerebellar Lobule VIIIa |
|  | 2.74 | 40 | -64 | -24 | Right Cerebellar Crus I |
| **DAN** |  |  |  |  |  |
| 149 | 4.08 | 42 | 34 | 18 | Right Middle Frontal Gyrus |
| **DMN** |  |  |  |  |  |
| 242 | 3.18 | -8 | 40 | 34 | Left Paracingulate Gyrus |
|  | 2.24 | -10 | 38 | 54 | Left Superior Frontal Gyrus |
| 197 | 3.79 | -8 | 56 | 24 | Left Superior Frontal Gyrus |
| **ECN** |  |  |  |  |  |
| 208 | 3.37 | -24 | 36 | 42 | Left Frontal Pole |
| 119 | 3.22 | 34 | 20 | 0 | Right Insular Cortex |
| **lFPN** |  |  |  |  |  |
| 226 | 3.25 | -6 | 42 | 48 | Left Superior Frontal Gyrus |
| 138 | 3.42 | -44 | 6 | 42 | Left Middle Frontal Gyrus |
| **rFPN** |  |  |  |  |  |
| 328 | 3.71 | 30 | 16 | 52 | Right Middle Frontal Gyrus |
|  | 2.66 | 46 | -2 | 40 | Right Precentral Gyrus |
| 229 | 3.97 | 68 | -32 | 0 | Right Middle Temporal Gyrus |
| 223 | 3.14 | 56 | -52 | 42 | Right Angular Gyrus |
| 176 | 3.60 | -50 | -56 | 44 | Left Angular Gyrus |
| 171 | 3.01 | 8 | 30 | 48 | Right Superior Frontal Gyrus |
| 143 | 3.03 | 40 | 36 | 28 | Right Frontal Pole |
| 138 | 3.35 | 54 | 34 | 0 | Right Inferior Frontal Gyrus, pars triangularis |
| 112 | 2.64 | 32 | -54 | 40 | Right Superior Parietal Lobule |
|  | 2.33 | 50 | -42 | 52 | Right Supramarginal Gyrus, posterior division |
| **LVN** |  |  |  |  |  |
| 419 | 3.86 | 12 | -68 | 18 | Right Cuneal Cortex |
|  | 2.78 | 20 | -84 | 30 | Right Lateral Occipital Cortex, superior division |
| 299 | 3.72 | 26 | -54 | -20 | Right Cerebellar Lobule VI |
|  | 3.51 | 44 | -42 | -18 | Right Temporal Occipital Fusiform Cortex |
| **MVN** |  |  |  |  |  |
| 192 | 2.98 | -2 | -62 | 8 | Left Lingual Gyrus |
| **SMN** |  |  |  |  |  |
| 121 | 3.21 | -8 | -24 | 46 | Left Precentral Gyrus |
| 115 | 3.01 | -34 | -10 | 56 | Left Precentral Gyrus |
| **Low-disability < HS** |  |  |  |  |  |
| **CBN** |  |  |  |  |  |
| 111 | 2.74 | -30 | -68 | -40 | Left Cerebellar Crus II |
|  |  |  |  |  |  |
| **Mild disability > HS** |  |  |  |  |  |
| **AUN** |  |  |  |  |  |
| 401 | 3.55 | 68 | -32 | 24 | Right Superior Temporal Gyrus, posterior division |
|  | 2.70 | 56 | -34 | 40 | Right Supramarginal Gyrus, anterior division |
|  | 2.52 | 50 | -32 | 12 | Right Planum Temporale |
| 107 | 3.07 | 46 | 20 | 2 | Right Frontal Operculum Cortex |
| **BGN** |  |  |  |  |  |
| 503 | 4.24 | 14 | 14 | -12 | Right Subcallosal Cortex |
|  | 2.33 | 24 | 6 | 10 | Right Putamen |
| 387 | 4.46 | -16 | 14 | -4 | Left Putamen |
| 316 | 3.46 | -14 | -40 | 0 | Left Hippocampus |
| 142 | 3.08 | 8 | -22 | 2 | Right Thalamus |
| **CBN** |  |  |  |  |  |
| 194 | 2.87 | -36 | -74 | -50 | Left Cerebellar Crus II |
| 115 | 3.08 | 14 | -60 | -26 | Right Cerebellar Lobule VI |
| **DAN** |  |  |  |  |  |
| 339 | 3.65 | -44 | -70 | 0 | Left Lateral Occipital Cortex, inferior division |
| 103 | 2.56 | 24 | -64 | 58 | Right Lateral Occipital Cortex, inferior division |
| 102 | 3.0 | 56 | -66 | -6 | Right Lateral Occipital Cortex, inferior division |
| **DMN** |  |  |  |  |  |
| 137 | 3.64 | -6 | 52 | 34 | Left Superior Frontal Gyrus |
| 121 | 3.09 | -2 | -60 | 18 | Left Precuneous Cortex |
| 106 | 2.68 | 10 | 58 | -6 | Right Frontal Pole |
| **ECN** |  |  |  |  |  |
| 248 | 4.40 | 32 | 18 | -2 | Right Insular Cortex |
| 224 | 2.83 | -30 | 24 | -12 | Left Frontal Orbital Cortex |
| 202 | 3.85 | -26 | 50 | 4 | Left Frontal Pole |
| 103 | 3.17 | -18 | 18 | 62 | Left Superior Frontal Gyrus |
| **lFPN** |  |  |  |  |  |
| 295 | 4.29 | -36 | 20 | 46 | Left Middle Frontal Gyrus |
| 104 | 3.73 | -30 | 0 | 60 | Left Middle Frontal Gyrus |
| **SMN** |  |  |  |  |  |
| 449 | 4.20 | -30 | -10 | 64 | Left Precentral Gyrus |
|  | 3.62 | -42 | -24 | 38 | Left Postcentral Gyrus |
| 140 | 4.41 | -8 | -10 | 46 | Left Juxtapositional Lobule Cortex (formerly Supplementary Motor Cortex) |
| **Mild-disability < HS** |  |  |  |  |  |
| **CBN** |  |  |  |  |  |
| 137 | 3.87 | 24 | -46 | -28 | Left Cerebellar Lobule VI |
| **lFPN** |  |  |  |  |  |
| 153 | 3.0 | -44 | 12 | 32 | Left Middle Frontal Gyrus |
| 119 | 3.10 | -46 | 42 | 24 | Left Frontal Pole |
| **rFPN** |  |  |  |  |  |
| 100 | 2.60 | 44 | 44 | 6 | Right Frontal Pole |
| **SMN** |  |  |  |  |  |
| 111 | 2.84 | -2 | -28 | 50 | Left Precentral Gyrus |
|  |  |  |  |  |  |
| **High-disability > HS** |  |  |  |  |  |
| **CBN** |  |  |  |  |  |
| 175 | 3.31 | 30 | -66 | -48 | Right Cerebellar Lobule VIIb |
| 125 | 2.83 | 28 | -40 | -38 | Right Cerebellar Lobule VI |
| 121 | 4.14 | -44 | -72 | -42 | Left Cerebellar Crus II |
| **DAN** |  |  |  |  |  |
| 216 | 3.86 | 58 | -38 | 32 | Right Supramarginal Gyrus, posterior division |
| 184 | 3.72 | -44 | -34 | 48 | Left Postcentral Gyrus |
| 110 | 2.61 | -26 | -70 | 44 | Left Lateral Occipital Cortex, superior division |
| 105 | 4.50 | 32 | -34 | 52 | Right Postcentral Gyrus |
| **DMN** |  |  |  |  |  |
| 269 | 3.68 | 16 | 42 | 38 | Right Frontal Pole |
|  | 3.44 | -8 | 42 | 34 | Left Superior Frontal Gyrus |
| 205 | 3.49 | -20 | 54 | 24 | Left Frontal Pole |
| 189 | 3.28 | 16 | 64 | 6 | Right Frontal Pole |
|  | 3.08 | 10 | 52 | 30 | Right Superior Frontal Gyrus |
| 102 | 2.68 | 40 | -36 | 50 | Right Superior Parietal Lobule |
| 100 | 2.78 | 4 | 52 | -4 | Right Paracingulate Gyrus |
| **ECN** |  |  |  |  |  |
| 224 | 3.32 | 32 | 50 | 36 | Right Frontal Pole |
|  | 1.83 | 36 | 32 | 26 | Right Middle Frontal Gyrus |
| 147 | 3.60 | -20 | 60 | 18 | Left Frontal Pole |
| 133 | 3.75 | -38 | 14 | -6 | Left Insular Cortex |
| 124 | 3.49 | 8 | 54 | 14 | Right Paracingulate Gyrus |
| **lFPN** |  |  |  |  |  |
| 255 | 5.17 | -40 | -74 | 24 | Left Lateral Occipital Cortex, superior division |
|  | 3.24 | -30 | -52 | 48 | Left Superior Parietal Lobule |
| 113 | 4.27 | -52 | -60 | 46 | Left Lateral Occipital Cortex, superior division |
| 110 | 3.43 | -38 | 44 | -6 | Left Frontal Pole |
| 108 | 3.86 | 0 | -30 | 44 | Cingulate Gyrus, posterior division |
|  | 2.40 | -4 | -36 | 24 | Left Cingulate Gyrus, posterior division |
| **SMN** |  |  |  |  |  |
| 255 | 2.96 | -40 | -40 | 62 | Left Superior Parietal Lobule |
|  | 2.35 | -44 | -26 | 48 | Left Postcentral Gyrus |
| 252 | 3.97 | 8 | -8 | 40 | Right Cingulate Gyrus, anterior division |
|  | 3.27 | -8 | -4 | 52 | Left Juxtapositional Lobule Cortex (formerly Supplementary Motor Cortex) |
| 212 | 4.18 | -34 | -12 | 62 | Left Precentral Gyrus |
| **High-disability < HS** |  |  |  |  |  |
| **AUN** |  |  |  |  |  |
| 160 | 4.46 | 36 | -8 | 10 | Right Insular Cortex |
| 106 | 3.08 | 64 | -8 | 16 | Right Postcentral Gyrus |
| 101 | 3.48 | 34 | 20 | 12 | Right Frontal Operculum Cortex |
| **CBN** |  |  |  |  |  |
| 126 | 3.50 | -8 | -26 | -22 | Left Brainstem |
|  | 3.0 | 12 | -24 | -28 | Right Brainstem |
| **rFPN** |  |  |  |  |  |
| 227 | 3.48 | 36 | -52 | 50 | Right Superior Parietal Lobule |
|  | 2.80 | 50 | -30 | 50 | Right Supramarginal Gyrus, anterior division |
| 163 | 3.73 | 14 | 48 | 46 | Right Frontal Pole |
| 119 | 3.14 | 46 | 0 | 58 | Right Precentral Gyrus |
| 110 | 2.89 | 58 | -50 | -12 | Right Inferior Temporal Gyrus, temporooccipital part |
| **MVN** |  |  |  |  |  |
| 180 | 3.08 | -6 | -64 | -6 | Left Lingual Gyrus |
|  | 2.19 | -12 | -78 | 14 | Left Intracalcarine Cortex |

**Supplementary Table 4**. Significant correlations between baseline within-network rsFC in pwMS groups and baseline motor and cognitive performance (p < 0.05, FDR corrected, minimum cluster extent set at 50 voxels). Refer to Supplementary Table 3 for a detailed explanation of the table layout.

|  |  | **MNI coordinates** | | |  |
| --- | --- | --- | --- | --- | --- |
| **Cluster size (voxels)** | **T** | **x** | **y** | **z** | **Cluster location (local maxima)** |
| **Low-disability** | | | | | |
| **LVN - T25FWT ↓** |  |  |  |  |  |
| 101 | 6.52 | 22 | -86 | 28 | Right Lateral Occipital Cortex, superior division |
| **rFPN - SDMT ↑** |  |  |  |  |  |
| 51 | 2.01 | 58 | -28 | -8 | Right Middle Temporal Gyrus, posterior division |
|  | 2.04 | 42 | -48 | 46 | Right Angular Gyrus |
|  |  |  |  |  |  |
| **Mild-disability** |  |  |  |  |  |
| **DMN - T25FWT ↑** |  |  |  |  |  |
| 54 | 7.73 | 8 | 60 | 2 | Right Frontal Pole |
| **AUN - SDMT ↓** |  |  |  |  |  |
| 67 | 4.79 | 62 | -42 | 36 | Right Supramarginal Gyrus, posterior division |
| 54 | 5.44 | 58 | -34 | 18 | Right Planum Temporale |
|  |  |  |  |  |  |
| **High-disability > HS** |  |  |  |  |  |
| **SMN - 9HPT-DH ↑** |  |  |  |  |  |
| 58 | 10.50 | -40 | -42 | 62 | Left Superior Parietal Lobule |
| 57 | 8.43 | -2 | -12 | 50 | Left Juxtapositional Lobule Cortex (formerly Supplementary Motor Cortex) |
| 46 | 10.10 | -34 | -16 | 66 | Left Precentral Gyrus |
|  |  |  |  |  |  |

#

#

**Supplementary Table 5**. Significant correlations between baseline within-network rsFC in pwMS groups and Δscores of motor and cognitive tests (p < 0.05, FDR corrected, minimum cluster extent set at 50 voxels). Refer to Supplementary Table 3 for a detailed explanation of the table layout.

|  |  | **MNI coordinates** | | |  |
| --- | --- | --- | --- | --- | --- |
| **Cluster size (voxels)** | **T** | **x** | **y** | **z** | **Cluster location (local maxima)** |
| **Low-disability** | | | | | |
| **AUN - Δ9HPT-DH ↓** |  |  |  |  |  |
| 60 | 2.04 | -52 | -10 | 2 | Left Heschl's Gyrus (includes H1 and H2) |
| **LVN - Δ9HPT-DH ↓** |  |  |  |  |  |
| 51 | 2.36 | 30 | -40 | -18 | Right Temporal Occipital Fusiform Cortex |
| **BGN - ΔPASAT3 ↑** |  |  |  |  |  |
| 71 | 3.12 | -10 | 12 | 10 | Left Caudate |
|  |  |  |  |  |  |
| **Mild-disability** |  |  |  |  |  |
| **BGN - Δ9HPT-DH ↑** |  |  |  |  |  |
| 83 | 3.99 | -28 | 8 | 0 | Left Putamen |
| 67 | 3.98 | -6 | -30 | 2 | Left Thalamus |
| **lFPN - Δ9HPT-DH ↑** |  |  |  |  |  |
| 58 | 4.75 | -40 | 34 | 40 | Left Middle Frontal Gyrus |
| **BGN - ΔT25FWT ↑** |  |  |  |  |  |
| 58 | 2.61 | 8 | 14 | -10 | Right Accumbens |
| **BGN - ΔPASAT3 ↓** |  |  |  |  |  |
| 74 | 2.32 | 28 | 8 | -10 | Right Putamen |
|  | 2.12 | 16 | 12 | 8 | Right Caudate |
| **DAN - ΔPASAT3 ↓** |  |  |  |  |  |
| 103 | 3.32 | -48 | -72 | -8 | Left Lateral Occipital Cortex, inferior division |
|  |  |  |  |  |  |

#

**Supplementary Table 6**. Resting-state networks (RSNs) showing significant group differences in longitudinal changes of functional connectivity (ΔFC = follow-up – baseline) among people with Multiple Sclerosis (pwMS) with low, mild, and high disability. (p < 0.05, FDR corrected, minimum cluster extent set at 100 voxels). Refer to Supplementary Table 3 for a detailed explanation of the table layout.

|  |  | **MNI coordinates** | | |  |
| --- | --- | --- | --- | --- | --- |
| **Cluster size (voxels)** | **T** | **x** | **y** | **z** | **Cluster location (local maxima)** |
| **Low-disability > Mild-disability** | | | | | |
| **AUN** |  |  |  |  |  |
| 148 | 3.47 | 60 | -26 | 16 | Right Planum Temporale |
| 126 | 4.05 | -50 | -8 | 6 | Left Central Opercular Cortex |
| 108 | 3.59 | 58 | -34 | 26 | Right Parietal Operculum Cortex |
| **BGN** |  |  |  |  |  |
| 296 | 4.34 | -25 | 7 | -8 | Left Putamen |
| **CBN** |  |  |  |  |  |
| 230 | 3.78 | 28 | -66 | -32 | Right Cerebellar Crus I |
| **DAN** |  |  |  |  |  |
| 102 | 3.74 | 30 | -74 | 36 | Right Lateral Occipital Cortex, superior division |
| **DMN** |  |  |  |  |  |
| 117 | 3.91 | -6 | 50 | 4 | Left Paracingulate Gyrus |
| **ECN** |  |  |  |  |  |
| 172 | 3.95 | 44 | 14 | -6 | Right Insular Cortex |
| **lFPN** |  |  |  |  |  |
| 147 | 4.90 | -42 | 28 | 38 | Left Middle Frontal Gyrus |
|  | 3.0 | -16 | 24 | 50 | Left Superior Frontal Gyrus |
| **rFPN** |  |  |  |  |  |
| 108 | 4.40 | 8 | 20 | 40 | Right Paracingulate Gyrus |
| **LVN** |  |  |  |  |  |
| 318 | 4.07 | -26 | -62 | -14 | Left Temporal Occipital Fusiform Cortex |
|  | 3.92 | -30 | -82 | -14 | Left Occipital Fusiform Gyrus |
|  | 3.63 | -20 | -68 | 12 | Left Intracalcarine Cortex |
| 176 | 4.08 | 42 | -70 | 0 | Right Lateral Occipital Cortex, inferior division |
| 102 | 3.23 | 32 | -54 | -8 | Right Temporal Occipital Fusiform Cortex |
| **MVN** |  |  |  |  |  |
| 327 | 3.94 | 14 | -74 | 8 | Right Intracalcarine Cortex |
|  | 3.37 | -6 | -88 | 14 | Left Supracalcarine Cortex |
| 106 | 3.43 | -10 | -68 | 18 | Left Precuneous Cortex |
| **SMN** |  |  |  |  |  |
| 317 | 3.60 | 46 | -14 | 54 | Right Precentral Gyrus |
|  | 2.94 | 38 | -36 | 64 | Right Postcentral Gyrus |
| 143 | 4.14 | -6 | -8 | 44 | Left Cingulate Gyrus, anterior division |
| 125 | 4.46 | -46 | -32 | 44 | Left Supramarginal Gyrus, anterior division |
|  |  |  |  |  |  |
| **Low-disability > High-disability** | | | | | |
| **AUN** |  |  |  |  |  |
| 121 | 3.92 | 44 | -26 | 18 | Right Parietal Operculum Cortex |
|  | 3.12 | 64 | -44 | 20 | Right Supramarginal Gyrus, posterior division |
| **BGN** |  |  |  |  |  |
| 201 | 3.57 | -24 | 11 | -8 | Left Putamen |
| **CBN** |  |  |  |  |  |
| 324 | 4.20 | 28 | -70 | -34 | Right Cerebellar Crus I |
| **DMN** |  |  |  |  |  |
| 106 | 4.11 | 52 | -62 | 24 | Right Lateral Occipital Cortex, superior division |
| **ECN** |  |  |  |  |  |
| 120 | 3.57 | 12 | 34 | 26 | Right Paracingulate Gyrus |
|  | 2.05 | -10 | 26 | 26 | Left Cingulate Gyrus |
| **LVN** |  |  |  |  |  |
| 274 | 4.68 | -26 | -54 | -4 | Left Lingual Gyrus |
|  | 4.25 | -30 | -80 | -18 | Left Occipital Fusiform Gyrus |
| **MVN** |  |  |  |  |  |
| 476 | 3.74 | -4 | -74 | 22 | Left Cuneal Cortex |
|  | 3.46 | 14 | -78 | 10 | Right Intracalcarine Cortex |
|  | 2.74 | -16 | -64 | 4 | Left Intracalcarine Cortex |
| **SMN** |  |  |  |  |  |
| 122 | 3.58 | -8 | -4 | 48 | Left Juxtapositional Lobule Cortex (formerly Supplementary Motor Cortex) |
|  |  |  |  |  |  |
| **High-disability > Mild-disability** | | | | | |
| **SMN** |  |  |  |  |  |
| 311 | 4.48 | 48 | -28 | 44 | Right Supramarginal Gyrus, anterior division |
|  | 2.11 | 44 | -12 | 56 | Right Precentral Gyrus |
| 122 | 3.56 | -30 | -28 | 66 | Left Postcentral Gyrus |
|  |  |  |  |  |  |

**Supplementary Table 7**. Voxel-wise main effects (ME) from the ANCOVA model showing significant longitudinal FC changes within resting-state networks (RSNs) for each disability group (p < 0.05, FDR corrected, minimum cluster extent set at 100 voxels). Refer to Supplementary Table 3 for a detailed explanation of the table layout.

|  |  | **MNI coordinates** | | |  |
| --- | --- | --- | --- | --- | --- |
| **Cluster size (voxels)** | **T** | **x** | **y** | **z** | **Cluster location (local maxima)** |
| **Low-disability – FC increments** | | | | | |
| **AUN** |  |  |  |  |  |
| 111 | 3.86 | 58 | -22 | 16 | Right Parietal Operculum Cortex |
|  | 2.89 | 42 | -14 | -4 | Right Planum Polare |
| 100 | 3.96 | -64 | -32 | 14 | Left Superior Temporal Gyrus, posterior division |
| **BGN** |  |  |  |  |  |
| 107 | 3.93 | 14 | -24 | 4 | Right Thalamus |
| **CBN** |  |  |  |  |  |
| 226 | 4.11 | 26 | -68 | -32 | Right Cerebellar Crus I |
| **DAN** |  |  |  |  |  |
| 289 | 4.74 | 30 | -74 | 36 | Right Lateral Occipital Cortex, superior division |
|  | 3.52 | 32 | -60 | 62 | Right Lateral Occipital Cortex, superior division |
| **ECN** |  |  |  |  |  |
| 113 | 4.03 | 12 | 34 | 24 | Right Paracingulate Gyrus |
| **lFPN** |  |  |  |  |  |
| 100 | 3.65 | -24 | 20 | 46 | Left Superior Frontal Gyrus |
| **rFPN** |  |  |  |  |  |
| 190 | 3.27 | 56 | 32 | 14 | Right Inferior Frontal Gyrus, pars triangularis |
| 173 | 3.30 | 44 | -68 | 38 | Right Lateral Occipital Cortex, superior division |
|  | 2.99 | 46 | -48 | 44 | Right Angular Gyrus |
| **LVN** |  |  |  |  |  |
| 339 | 4.58 | -30 | -60 | -12 | Left Temporal Occipital Fusiform Cortex |
| 264 | 3.62 | 30 | -54 | -10 | Right Temporal Occipital Fusiform Cortex |
| 102 | 3.55 | -4 | -64 | -4 | Left Lingual Gyrus |
| 101 | 3.07 | -10 | -84 | 32 | Left Cuneal Cortex |
| **MVN** |  |  |  |  |  |
| 949 | 4.48 | 8 | -76 | 6 | Right Intracalcarine Cortex |
|  | 3.72 | -16 | -64 | 4 | Left Intracalcarine Cortex |
|  |  |  |  |  |  |
| **Mild-disability – FC increments** | | | | | |
| **FPR** |  |  |  |  |  |
| 144 | 3.86 | 38 | 32 | 38 | Right Middle Frontal Gyrus |
| **Mild-disability – FC decrements** | | | | | |
| **BGN** |  |  |  |  |  |
| 237 | 2.93 | -10 | 20 | -10 | Subcallosal Cortex |
| 158 | 3.89 | 24 | 6 | -12 | Right Putamen |
|  | 3.03 | 24 | 2 | 8 | Right Putamen |
| **CBN** |  |  |  |  |  |
| 167 | 3.78 | 44 | -62 | -28 | Right Cerebellar Crus I |
| **DAN** |  |  |  |  |  |
| 111 | 3.68 | -46 | -64 | -14 | Left Inferior Temporal Gyrus, temporooccipital part |
| **DMN** |  |  |  |  |  |
| 103 | 3.21 | -8 | 56 | 32 | Left Frontal Pole |
| **ECN** |  |  |  |  |  |
| 171 | 3.34 | 36 | 14 | -8 | Right Insular Cortex |
|  | 2.17 | 56 | 16 | 4 | Right Inferior Frontal Gyrus, pars opercularis |
| **lFPN** |  |  |  |  |  |
| 131 | 4.59 | -42 | 28 | 38 | Left Middle Frontal Gyrus |
| 130 | 3.25 | 0 | 20 | 48 | Paracingulate Gyrus |
|  | 2.88 | -16 | 26 | 62 | Left Superior Frontal Gyrus |
| **LVN** |  |  |  |  |  |
| 264 | 3.32 | 54 | -64 | -10 | Right Lateral Occipital Cortex, inferior division |
| **SMN** |  |  |  |  |  |
| 363 | 3.30 | 44 | -18 | 52 | Right Postcentral Gyrus |
| 281 | 4.12 | -46 | -32 | 42 | Left Supramarginal Gyrus, anterior division |
| 211 | 4.23 | -4 | -8 | 44 | Left Cingulate Gyrus, anterior division |
| 137 | 3.29 | -30 | -18 | 68 | Left Precentral Gyrus |
|  |  |  |  |  |  |
| **High-disability – FC increments** | | | | | |
| **SMN** |  |  |  |  |  |
| 220 | 4.79 | 48 | -24 | 54 | Right Postcentral Gyrus |
| 152 | 3.76 | -30 | -30 | 66 | Left Postcentral Gyrus |
| **High-disability – FC decrements** | | | | | |
| **AUN** |  |  |  |  |  |
| 109 | 3.48 | 68 | -34 | 24 | Right Superior Temporal Gyrus, posterior division |
| **BGN** |  |  |  |  |  |
| 268 | 3.21 | -28 | 6 | 6 | Left Putamen |
| 202 | 2.97 | 20 | 8 | -2 | Right Putamen |
| **CBN** |  |  |  |  |  |
| 438 | 3.68 | 28 | -70 | -34 | Right Cerebellar Crus I |
|  | 3.27 | 8 | -80 | -44 | Right Cerebellar Crus II |
| **DAN** |  |  |  |  |  |
| 101 | 2.96 | -36 | -54 | 40 | Left Angular Gyrus |
| **DMN** |  |  |  |  |  |
| 191 | 3.95 | 60 | -64 | 20 | Right Lateral Occipital Cortex, superior division |
| 141 | 3.19 | 0 | 54 | 14 | Paracingulate Gyrus |
| **ECN** |  |  |  |  |  |
| 199 | 3.14 | 4 | 50 | -4 | Right Paracingulate Gyrus |
|  | 2.75 | 10 | 34 | 18 | Right Cingulate Gyrus, anterior division |
| **rFPN** |  |  |  |  |  |
| 174 | 3.12 | 44 | -50 | 38 | Right Angular Gyrus |
| 140 | 4.17 | 62 | -8 | 0 | Right Planum Temporale |
| **LVN** |  |  |  |  |  |
| 109 | 3.37 | -28 | -80 | -18 | Left Occipital Fusiform Gyrus |
| **MVN** |  |  |  |  |  |
| 107 | 3.02 | 12 | -92 | -4 | Right Occipital Pole |
| 104 | 3.57 | -4 | -74 | 22 | Left Cuneal Cortex |
| **SMN** |  |  |  |  |  |
| 199 | 3.27 | -4 | -12 | 52 | Left Juxtapositional Lobule Cortex (formerly Supplementary Motor Cortex) |
| 106 | 3.19 | -26 | -8 | 52 | Left Precentral Gyrus |
|  |  |  |  |  |  |

**Supplementary Table 8**. Significant correlations between ΔFC maps and Δscores of motor and cognitive tests (p < 0.05, FDR corrected, minimum cluster extent set at 50 voxels). Refer to Supplementary Table 3 for a detailed explanation of the table layout.

|  |  | **MNI coordinates** | | |  |
| --- | --- | --- | --- | --- | --- |
| **Cluster size (voxels)** | **T** | **x** | **y** | **z** | **Cluster location (local maxima)** |
| **Low-disability** | | | | | |
| **DAN - Δ9HPT-DH ↑** |  |  |  |  |  |
| 68 | 5.0 | 32 | -72 | 34 | Right Lateral Occipital Cortex, superior division |
| **MVN - Δ9HPT-NDH ↑** |  |  |  |  |  |
| 56 | 3.0 | 0 | -76 | 8 | Intracalcarine Cortex |
| **DAN - ΔT25FWT ↓** |  |  |  |  |  |
| 68 | 3.81 | 32 | -68 | 40 | Right Lateral Occipital Cortex, superior division |
| **LVN - ΔT25FWT ↓** |  |  |  |  |  |
| 74 | 5.22 | -26 | -62 | -10 | Left Temporal Occipital Fusiform Cortex |
| **MVN - ΔT25FWT ↓** |  |  |  |  |  |
| 77 | 3.05 | 14 | -78 | 2 | Right Intracalcarine Cortex |
|  |  |  |  |  |  |
| **Mild-disability** |  |  |  |  |  |
| **CBN - Δ9HPT-NDH ↓** |  |  |  |  |  |
| 53 | 4.05 | 28 | -64 | -30 | Right Cerebellar Lobule VI |
| **CBN - ΔT25FWT ↓** |  |  |  |  |  |
| 55 | 5.98 | 36 | -64 | -32 | Right Cerebellar Crus I |
| **ECN - ΔT25FWT ↓** |  |  |  |  |  |
| 57 | 3.37 | 50 | 18 | -4 | Right Frontal Operculum Cortex |
| **SMN - PBVC ↑** |  |  |  |  |  |
| 50 | 3.40 | 38 | -16 | 46 | Right Precentral Gyrus |
|  |  |  |  |  |  |
| **High-disability** |  |  |  |  |  |
| **SMN - Δ9HPT-DH ↑** |  |  |  |  |  |
| 53 | 3.52 | 44 | -24 | 50 | Right Postcentral Gyrus |
|  |  |  |  |  |  |

# **Supplementary Figures**

**Supplementary Figure 1.** Resting-state networks (RSNs) showing the highest spatial correlation coefficients with RSN templates and used for dual regression analysis: auditory (AUN, r=0.63), basal ganglia (BGN, r=0.53), cerebellar (CBN, r=0.45), dorsal attention (DAN, r=0.55), default mode (DMN, r=0.58), executive control (ECN, r=0.57), left and right frontoparietal (lFPN, rFPN, r=0.64 and r=0.64, respectively), lateral visual (LVN, r=0.54), medial visual (MVN, r=0.73), and sensorimotor (SMN, r=0.53) network. This figure shows sagittal, coronal, and axial slices for the RSNs detected, overlaid onto the MNI152 standard brain. RSNs are shown in FSL red-yellow color encoding using a 3 < z-score < 10 threshold window.

**
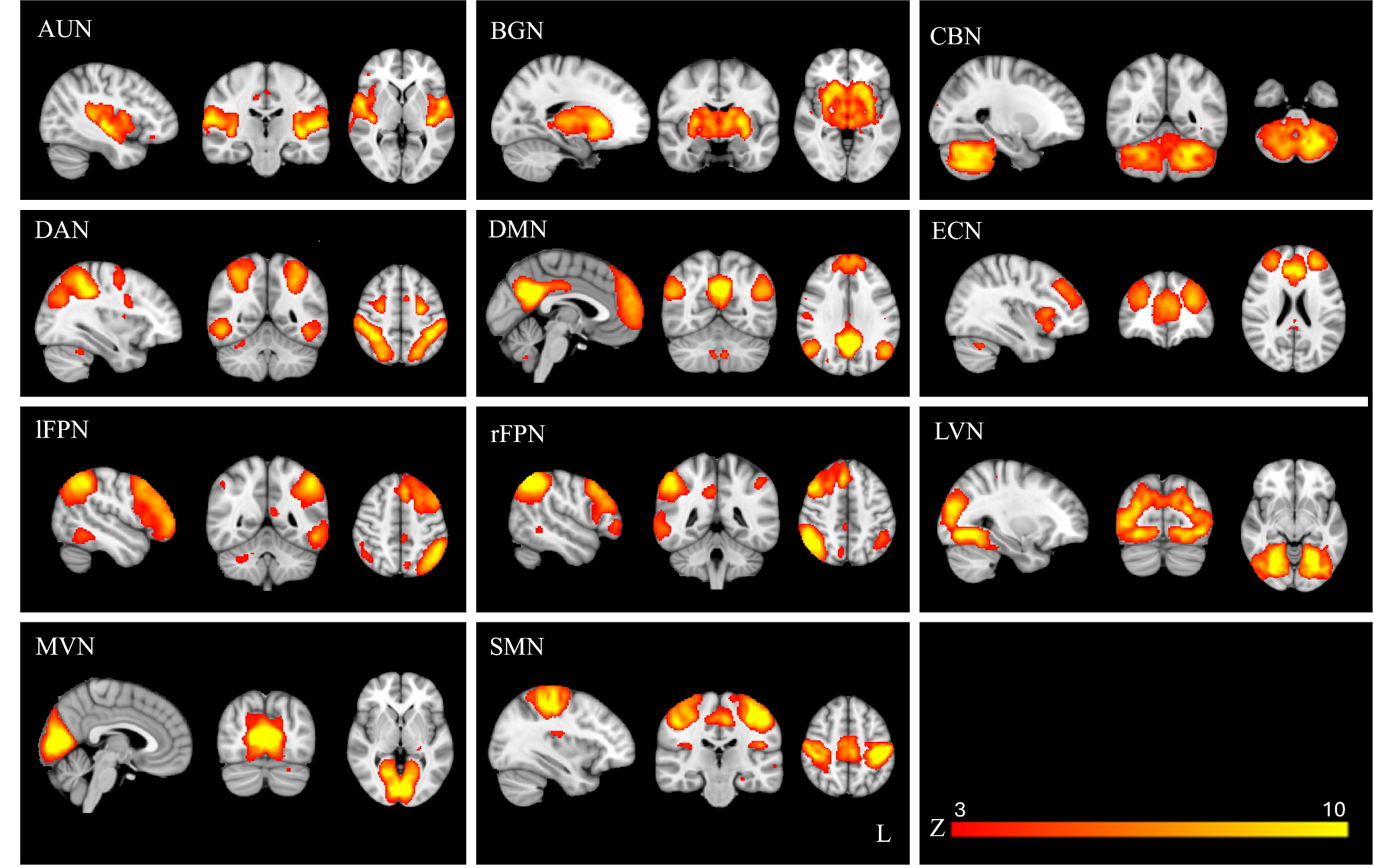
**

**Supplementary Figure 2.**  Significant correlations between baseline within-network rsFC in pwMS groups and baseline motor and cognitive performance (p < 0.05, FDR corrected). Results for each RSN are overlaid onto the corresponding network (green) in the MNI152 standard brain. Red-yellow and blue-light blue color bars represent t values.

**
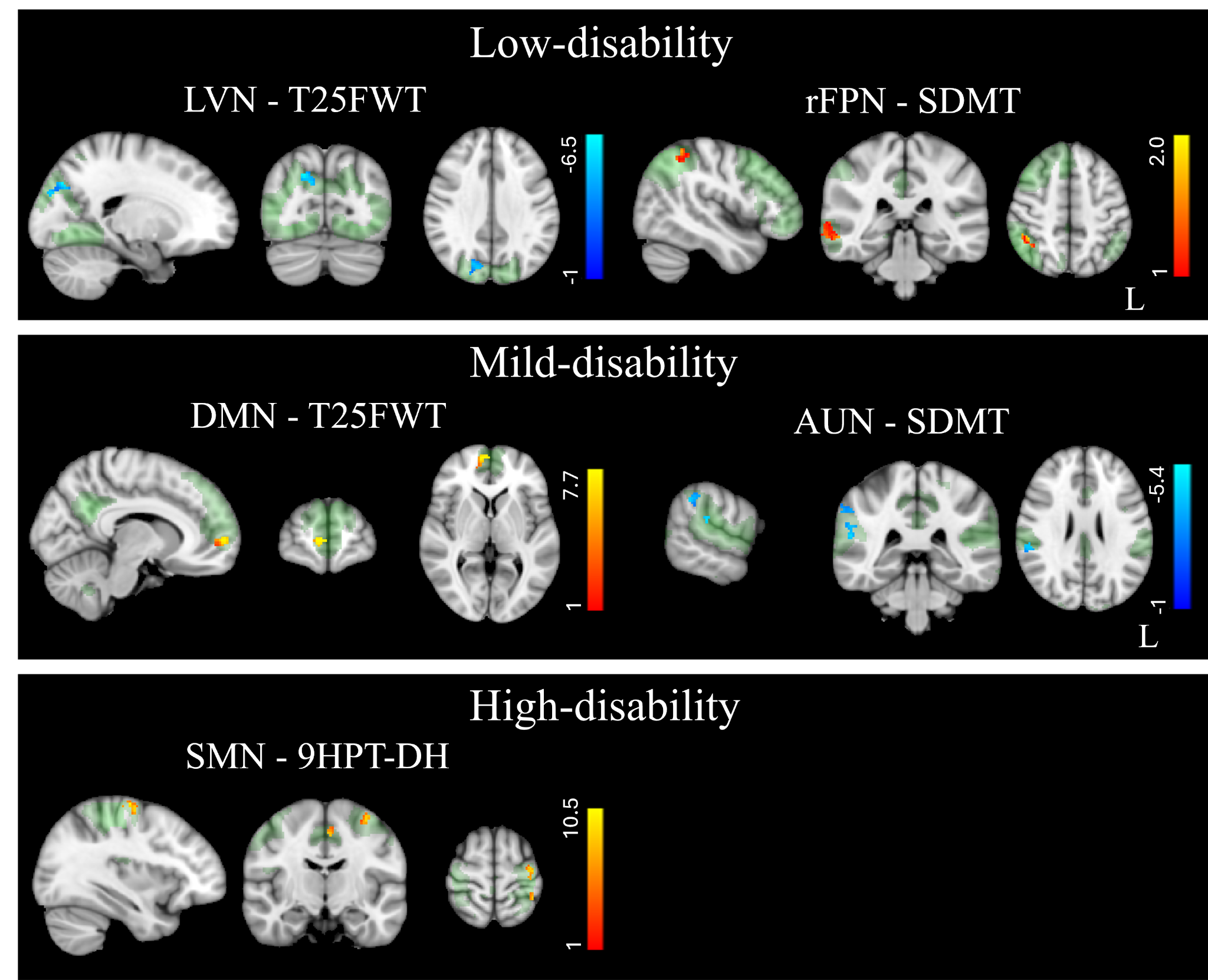
**

**Supplementary Figure 3.**  Significant correlations between baseline within-network rsFC in pwMS groups and Δscores of motor and cognitive tests (p < 0.05, FDR corrected). Results for each RSN are overlaid onto the corresponding network (green) in the MNI152 standard brain. Red-yellow and blue-light blue color bars represent t values.

**
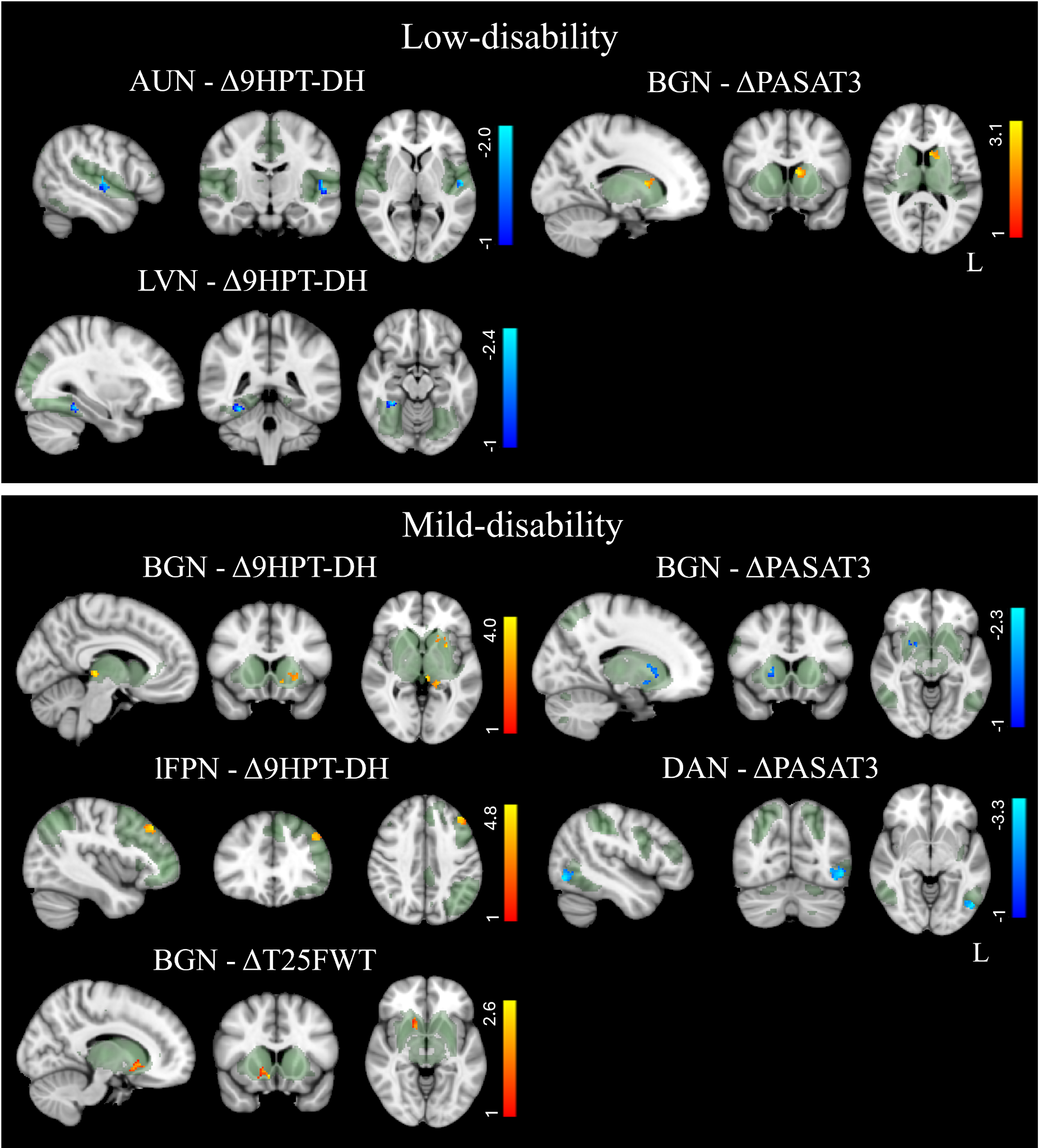
**

**Supplementary Figure 4.**  Significant correlations between ΔFC maps and Δscores of behavioral tests (p < 0.05, FDR corrected). Results for each RSN are overlaid onto the corresponding network (green) in the MNI152 standard brain. Red-yellow and blue-light blue color bars represent t values.

**
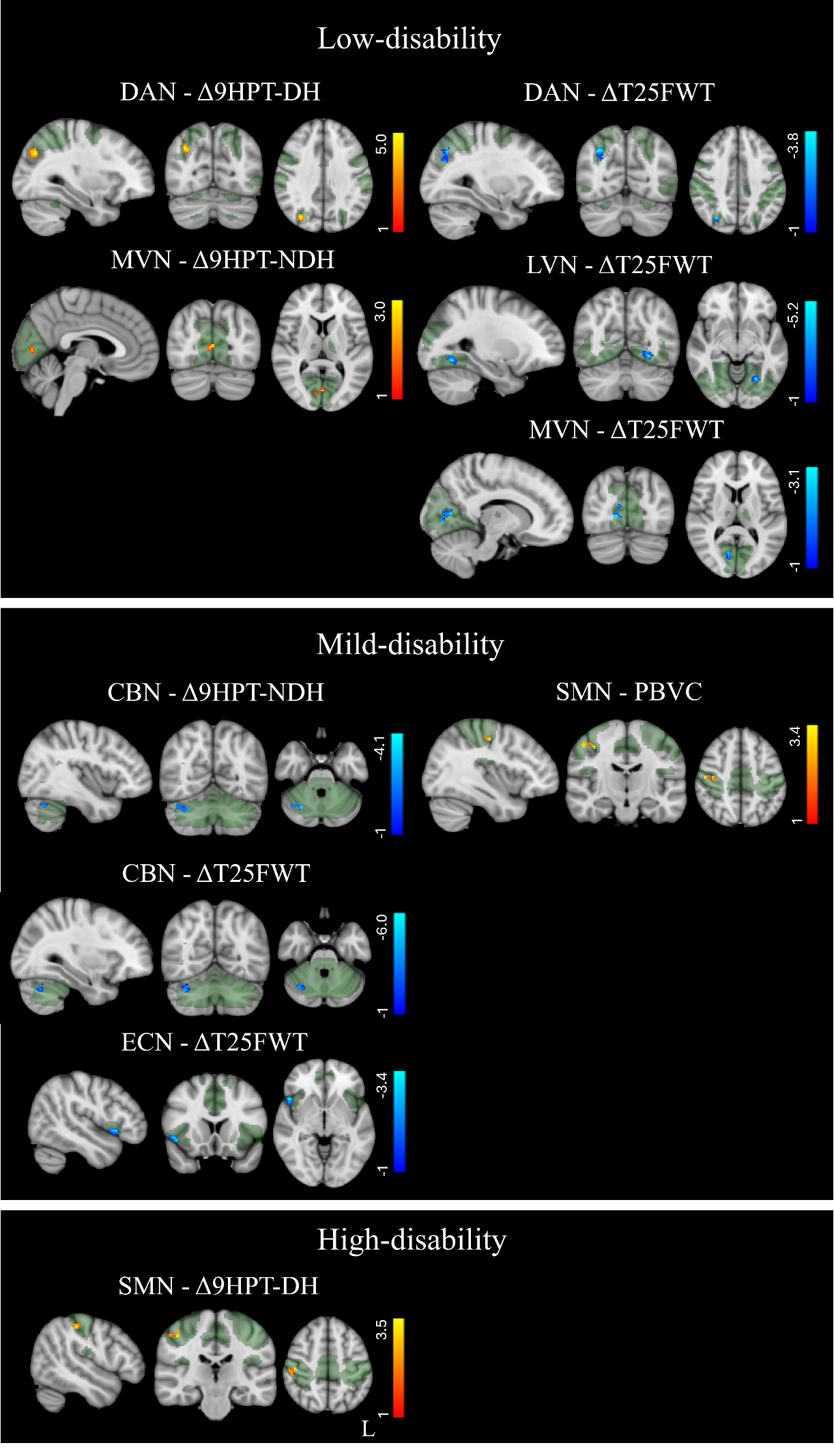
**

# **References**

[1.](https://www.zotero.org/google-docs/?Wpg2Vz)  [Esteban O, Ross B, Markiewicz CJ, et al (2018) fMRIPrep: a robust preprocessing pipeline for functional MRI (Version 23.0.2). Zenodo. https://doi.org/10.5281/zenodo.7863421](https://www.zotero.org/google-docs/?Wpg2Vz)

[2.](https://www.zotero.org/google-docs/?Wpg2Vz)  [Gorgolewski K, Burns CD, Madison C, et al (2011) Nipype: A Flexible, Lightweight and Extensible Neuroimaging Data Processing Framework in Python. Front Neuroinform 5:. https://doi.org/10.3389/fninf.2011.00013](https://www.zotero.org/google-docs/?Wpg2Vz)

[3.](https://www.zotero.org/google-docs/?Wpg2Vz)  [Gorgolewski K (2018) “Nipype.” Software. Zenodo. https://doi.org/10.5281/zenodo.596855](https://www.zotero.org/google-docs/?Wpg2Vz)

[4.](https://www.zotero.org/google-docs/?Wpg2Vz)  [Tustison NJ, Avants BB, Cook PA, et al (2010) N4ITK: Improved N3 Bias Correction. IEEE Transactions on Medical Imaging 29:1310–1320. https://doi.org/10.1109/TMI.2010.2046908](https://www.zotero.org/google-docs/?Wpg2Vz)

[5.](https://www.zotero.org/google-docs/?Wpg2Vz)  [Avants BB, Epstein CL, Grossman M, Gee JC (2008) Symmetric diffeomorphic image registration with cross-correlation: Evaluating automated labeling of elderly and neurodegenerative brain. Medical Image Analysis 12:26–41. https://doi.org/10.1016/j.media.2007.06.004](https://www.zotero.org/google-docs/?Wpg2Vz)

[6.](https://www.zotero.org/google-docs/?Wpg2Vz)  [Zhang Y, Brady M, Smith S (2001) Segmentation of brain MR images through a hidden Markov random field model and the expectation-maximization algorithm. IEEE Transactions on Medical Imaging 20:45–57. https://doi.org/10.1109/42.906424](https://www.zotero.org/google-docs/?Wpg2Vz)

[7.](https://www.zotero.org/google-docs/?Wpg2Vz)  [Fonov V, Evans A, McKinstry R, et al (2009) Unbiased nonlinear average age-appropriate brain templates from birth to adulthood. NeuroImage 47:S102. https://doi.org/10.1016/S1053-8119(09)70884-5](https://www.zotero.org/google-docs/?Wpg2Vz)

[8.](https://www.zotero.org/google-docs/?Wpg2Vz)  [Evans AC, Janke AL, Collins DL, Baillet S (2012) Brain templates and atlases. NeuroImage 62:911–922. https://doi.org/10.1016/j.neuroimage.2012.01.024](https://www.zotero.org/google-docs/?Wpg2Vz)

[9.](https://www.zotero.org/google-docs/?Wpg2Vz)  [Jenkinson M, Smith S (2001) A global optimisation method for robust affine registration of brain images. Medical Image Analysis 5:143–156. https://doi.org/10.1016/S1361-8415(01)00036-6](https://www.zotero.org/google-docs/?Wpg2Vz)

[10.](https://www.zotero.org/google-docs/?Wpg2Vz)  [Greve DN, Fischl B (2009) Accurate and robust brain image alignment using boundary-based registration. Neuroimage 48:63–72. https://doi.org/10.1016/j.neuroimage.2009.06.060](https://www.zotero.org/google-docs/?Wpg2Vz)

[11.](https://www.zotero.org/google-docs/?Wpg2Vz)  [Jenkinson M, Bannister P, Brady M, Smith S (2002) Improved optimization for the robust and accurate linear registration and motion correction of brain images. Neuroimage 17:825–841. https://doi.org/10.1016/s1053-8119(02)91132-8](https://www.zotero.org/google-docs/?Wpg2Vz)

[12.](https://www.zotero.org/google-docs/?Wpg2Vz)  [Cox RW, Hyde JS (1997) Software tools for analysis and visualization of fMRI data. NMR Biomed 10:171–178. https://doi.org/10.1002/(sici)1099-1492(199706/08)10:4/5%253C171::aid-nbm453%253E3.0.co;2-l](https://www.zotero.org/google-docs/?Wpg2Vz)

[13.](https://www.zotero.org/google-docs/?Wpg2Vz)  [Pruim RHR, Mennes M, van Rooij D, et al (2015) ICA-AROMA: A robust ICA-based strategy for removing motion artifacts from fMRI data. Neuroimage 112:267–277. https://doi.org/10.1016/j.neuroimage.2015.02.064](https://www.zotero.org/google-docs/?Wpg2Vz)
